# Molecular and Antigenic Characterization of Avian H9N2 Viruses in Southern China

**DOI:** 10.1128/spectrum.00822-21

**Published:** 2022-01-12

**Authors:** Wanwan Yan, Hongrui Cui, Marc Engelsma, Nancy Beerens, Monique M. van Oers, Mart C. M. de Jong, Xuesong Li, Qinfang Liu, Jianmei Yang, Qiaoyang Teng, Zejun Li

**Affiliations:** a Shanghai Veterinary Research Institute, Chinese Academy of Agriculture Sciences, Shanghai, China; b Quantitative Veterinary Epidemiology, Animal Sciences Group, Wageningen University & Research, Lelystad, the Netherlands; c Wageningen Bioveterinary Research, Wageningen University & Research, Lelystad, the Netherlands; d Laboratory of Virology, Plant Science Group, Wageningen University & Research, Lelystad, the Netherlands; US Food and Drug Administration

**Keywords:** phylogenic analysis, antigenic change, LPAI, H9N2

## Abstract

The H9N2 subtype avian influenza virus (AIV) has become endemic in poultry globally; however due to its low pathogenicity, it is not under primary surveillance and control in many countries. Recent reports of human infection caused by H9N2 AIV has increased public concern. This study investigated the genetic and antigenic characteristics of H9N2 AIV isolated from local markets in nine provinces in Southern China from 2013 to 2018. We detected an increasing annual isolation rate of H9N2 AIV. Phylogenetic analyses of hemagglutinin (HA) genes suggests that isolated strains were rooted in BJ94 lineage but have evolved into new subgroups (II and III), which derived from subgroup I. The estimated substitution rate of the subgroup III strains was 6.23 × 10^−3^ substitutions/site/year, which was 1.5-fold faster than that of the average H9N2 HA rate (3.95 × 10^−3^ substitutions/site/year). Based on the antigenic distances, subgroup II and III strains resulted in two clear antigenic clusters 2 and 3, separated from the vaccine strain F98, cluster 1. New antigenic properties of subgroup III viruses were associated with 11 amino acid changes in the HA protein, suggesting antigenic drift in H9N2 viruses. Our phylogenetic and antigenic analyses of the H9N2 strains circulating in local markets in Southern China provide new insights on the antigenic diversification of H9N2 viruses.

**IMPORTANCE** The H9N2 low pathogenicity avian influenza (LPAI) virus has become endemic in poultry globally. In several Asian countries, vaccination against H9N2 avian influenza virus (AIV) was approved to reduce economic losses in the poultry industry. However, surveillance programs initiated after the introduction of vaccination identified the persistence of H9N2 AIV in poultry (especially in chicken in South Korea and China). Recent reports of human infection caused by H9N2 AIV has increased public concern. Surveillance of H9N2 circulating in poultry in the fields or markets was essential to update the vaccination strategies. This study investigated the genetic and antigenic characteristics of H9N2 AIVs isolated from local markets in nine provinces in Southern China from 2013 to 2018. The discovery of mutations in the hemagglutinin (HA) gene that result in antigenic changes provides a baseline reference for evolutionary studies of H9N2 viruses and vaccination strategies in poultry.

## INTRODUCTION

Currently, H9N2 avian influenza virus (AIV) is one of the predominant subtypes (together with H5 and H7 subtype AIVs) circulating in both wild birds and poultry (1, 2). Ever since the first isolation reported in the United States in 1966, the H9N2 subtype AIV has become endemic in many countries across different continents, including Africa, Asia, and Europe (3, 4). Despite the direct economic loss in the poultry industry over the past decade, H9N2 AIV also has a potential threat for public health by providing inner genes for reassortment. Recent reports of human infection caused by H9N2 AIV has increased public concerns (5–8).

Surveillance of H9N2 AIV is carried out in many countries, especially China and other Asian countries in which live poultry markets (LPMs) are potential areas for human-poultry transmission or reassortment (9, 10). Genetic analyses of H9N2 AIV show two main phylogenic branches, the Eurasian branch and the American branch. The Eurasian branch consists of at least three stable poultry lineages, G1 (A/quail/Hong Kong/G1/1997), BJ94 (A/chicken/Beijing/1/94), and Y439/Korean (A/chicken/Hong Kong/Y439/1997) (11, 12). However, research shows that the dominant H9N2 AIV lineage differs depending on which region of endemicity is considered. The BJ-94-like lineage is suggested to be dominant in poultry on mainland China (11). Li et al. reported that hemagglutinin (HA) genes of some H9N2 isolates from chicken and other hosts between 2009 and 2011 were derived mainly from the Y280-like lineage (13). In addition, Yang et al. reported G9-like H9N2 viruses as being isolated primarily in mainland China (14). The Y280 and G9 lineage developed from the early BJ94 lineage has been detected throughout China since 1994 (4).

In addition to surveillance programs, some countries have also implemented poultry vaccination to prevent H9N2 AIV from becoming endemic (15–18). After 1998, the vaccination program was implemented in China with inactivated H9N2 vaccines based on the A/chicken/Shanghai/F/98 strain (16) and A/chicken/Shandong/6/96 strain (19). Korea started the national vaccination strategy using inactivated H9N2 vaccine in 2007 (20). However, due to the rapid mutation of RNA viruses, AIVs undergo antigenic drift under immune pressure from the large-scale and long-term use of vaccines. Lessons learned from the failure of vaccines in prohibiting virus shedding and transmission of AIV (21, 22) show the need for regular updates of vaccine strains. For example, the A/chicken/Shanghai/F/98 strain was found to be entirely different from the G1 lineage directly related to human infection in Hong Kong, which was then selected for a commercial poultry vaccine used on mainland China (23, 24). Thus, surveillance programs providing information on the genetic characteristics of H9N2 AIV that relate to potential antigenic changes contribute to better management. Additionally, the continuous circulation of H9N2 in vaccinated poultry not only hints at an antigenic drift and/or clade replacement by vaccine but also introduces doubt on whether inactivated vaccines are effective at stopping transmission of low pathogenicity avian influenza (LPAI) H9N2 in poultry. Our previous research (25) indicated the high possibility of transmission in vaccinated chickens with inactivated vaccines. This, consequently, might lead genetic changes toward antigenic mutations. Research that combines genetic mutations with antigenic changes is needed to better understand the molecular determinants of H9 antigenicity.

Since 2003, monitoring programs of LPAI H9N2 in Southern China were conducted by the Research Team of the Etiologic Ecology of Animal Influenza and Avian Emerging Viral Disease in Shanghai Veterinary Research Institute (SHVRI). Swab samples from domestic poultry (chickens, ducks, pigeons, geese) were collected annually in the local markets in Southern China. The most recent samples collected from 2013 to 2018 showed an increasing annual isolation rate of H9N2 AIV in Southern China. The phylogenetic and antigenic analyses of the H9N2 isolates were combined to identify a common clustering change. The most recent isolates clustered together but were far from the vaccine strains. Isolated strains forming a novel antigenic group in antigenic assays also clustered in a new subgroup in the phylogenetic tree. The evolution rate of strains in the new antigenic group was estimated to be higher than the average rate of H9N2 in China. Moreover, amino acid changes on HA protein indicating potential antigenic sites provide new evidence for the molecular evolution of H9N2.

## RESULTS

### Epidemiology information of samples from Southern China during 2013 to 2018.

**FIG 1:**
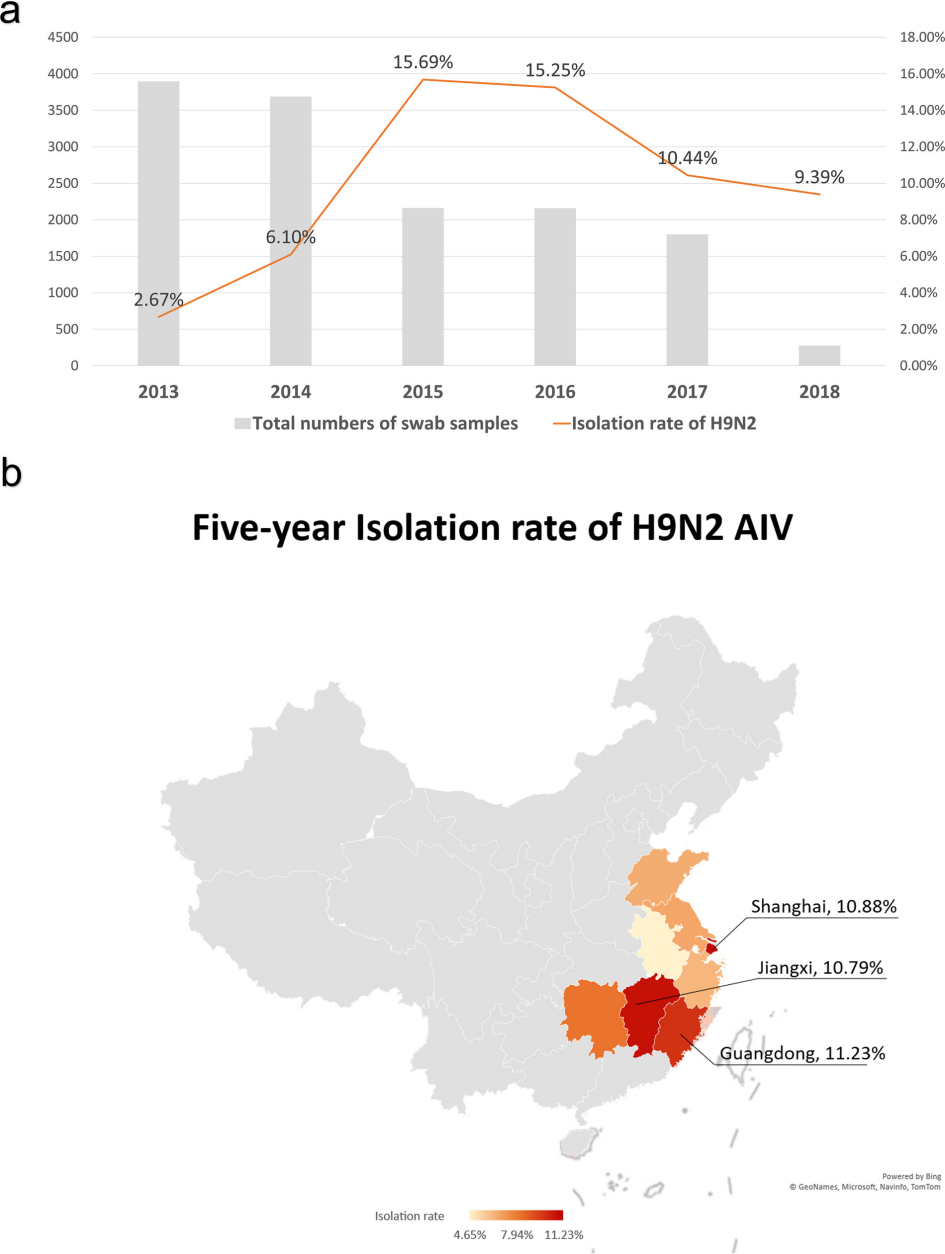
Isolation information of H9N2 in nine provinces in Southern China. (a) Annual isolation rate of H9N2 from all nine provinces in Southern China. The total height of every bar in the histogram is the total number of AIV-positive samples collected per year from 2013 to 2018. The top yellow part of the bar is the number of detected H9N2-positive samples. The gray line shows the isolation rate of H9N2 over the years. (b) The isolation rate of H9N2 in each province in the period from 2013 to 2018. Based on the 5-year total isolation rate, the provinces are color marked: dark red shows the highest isolation rate in percentages in the period under study. The data from the top three areas were listed. No samples were collected in gray areas.

To monitor the prevalence of H9N2 AIV in Southern China, we collected oropharynx and cloaca samples of poultry from LPMs in nine cities of different provinces in Southern China. From 2013 to 2018, a total of 13,981 samples were collected from chickens (9,363 samples), ducks (4,505 samples), geese (81 samples), and pigeons (32 samples) in different LPMs (Table 1). H4, H6, H9, and H10 subtypes of AIVs were identified. The total isolation rate of the H9N2 subtype accounted for the majority of the 1,549 avian influenza-positive samples, with a ratio of 78.18% (1,211/1,549) over the whole period. The isolation rates of H9N2 AIV in 2013, 2014, 2015, 2016, 2017, and 2018 were 2.67% (104/3,895), 6.10% (225/3,689), 15.69% (339/2,161), 15.25% (329/2,158), 10.44% (188/1,801), and 9.39% (26/277), respectively (Fig. 1a; Table 1). At a regional perspective, the H9N2 AIV isolation rate is higher in the three provinces and cities: Guangzhou, 11.23%; Shanghai, 10.88%; Jiangxi 10.79% (Fig. 1b). In total, 113 H9 strains were isolated from 2013 to 2018 in this study, while 100 H9 viruses were isolated from chickens and 13 H9 viruses were isolated from ducks. No H9 strains were isolated from geese or pigeons in this study.

**TABLE 1:** H9N2 isolation rate out of the AIV-positive samples collected from field markets in Southern China from 2013 to 2018

	Isolation yr^*a*^																	
	2013			2014			2015			2016			2017			2018		
City	Total^*b*^	AIV^*c*^	H9N2^*d*^	Total^*b*^	AIV^*c*^	H9N2^*d*^	Total^*b*^	AIV^*c*^	H9N2^*d*^	Total^*b*^	AIV^*c*^	H9N2^*d*^	Total^*b*^	AIV^*c*^	H9N2^*d*^	Total^*b*^	AIV^*c*^	H9N2^*d*^
Guangzhou	1232	75	59	861	79	47	632	133	120	606	181	171	302	14	11	NA	NA	NA
Fujian	478	16	11	339	46	24	184	42	36	140	25	22	156	29	25	95	21	20
Jiangxi	NA	NA	NA	386	55	30	244	32	26	300	30	25	256	47	47	NA	NA	NA
Zhejiang	154	2	2	158	10	4	130	27	22	NA	NA	NA	NA	NA	NA	NA	NA	NA
Shanghai	288	24	21	273	35	25	61	3	2	32^*e*^	32	17	155	24	23	NA	NA	NA
Anhui	446	6	6	359	36	19	330	48	31	360	0	0	313	37	28	NA	NA	NA
Jiangsu	337	0	0	516	30	18	281	72	54	305	31	26	419	48	37	158	1	1
Shandong	514	1	0	459	42	33	21^*e*^	19	15	207	32	31	NA	NA	NA	NA	NA	NA
Hunan	446	11	5	338	47	25	278	35	33	208	38	37	200	27	17	24	6	5
Total	3895	135	104	3689	380	225	2161	411	339	2158	369	329	1801	226	188	277	28	26

a NA, no data.

b Number of total swab samples collected from ducks/chickens in the market.

c Total number of AIV-positive samples.

d Total number of H9N2-positive samples.

e Samples from sick poultry with flu-like symptoms that tested positive in the HA assay.

### The phylogenic tree of HA genes.

**FIG 2:**
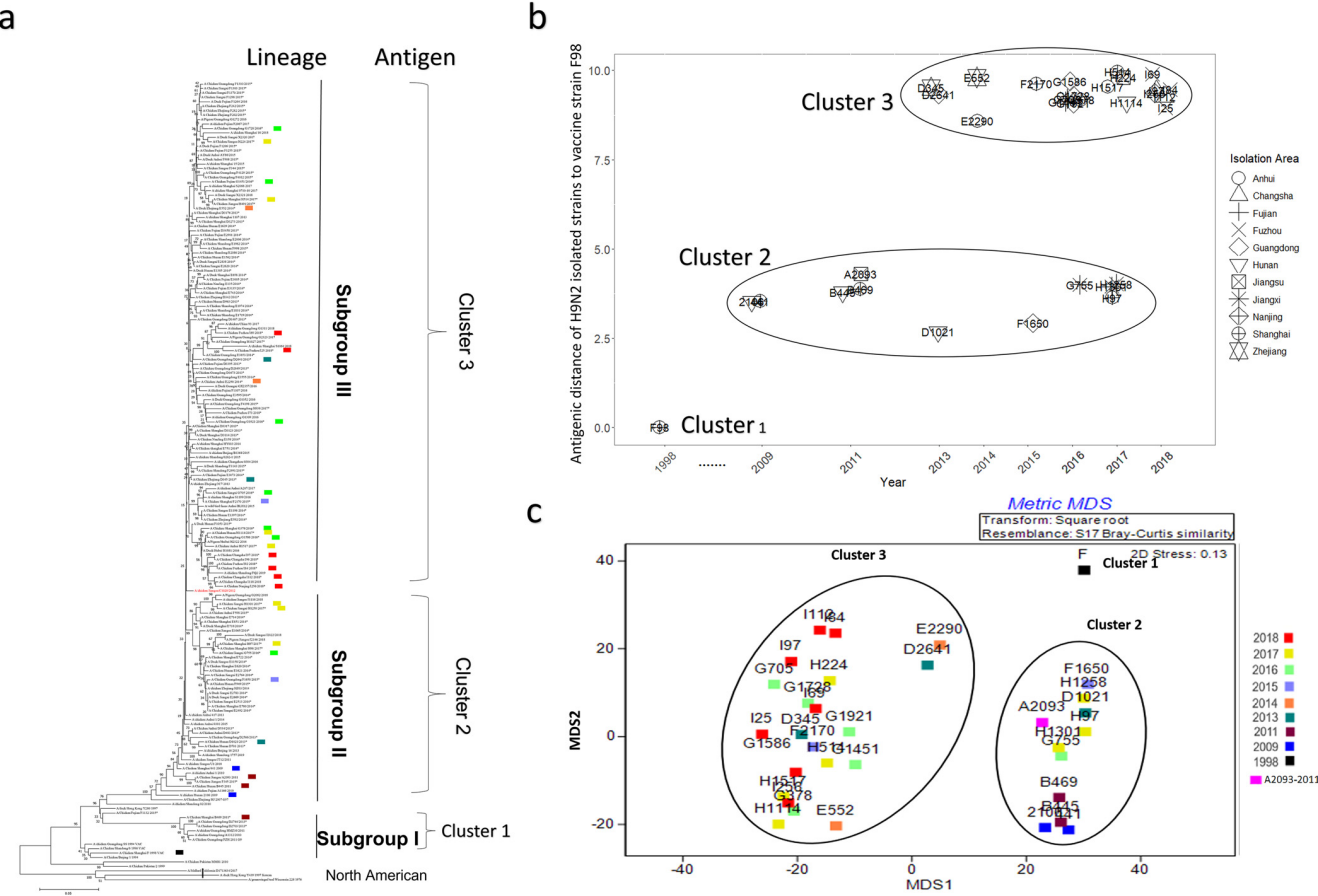
(a to c) Neighbor-joining tree of the HA gene (a) and antigenic distance of H9N2 isolated strains to vaccine strain F98 (b and c). H9N2 strains were isolated from 2013 to 2018 and are represented as an asterisk (*). The isolated viruses were selected for antigenic analysis and are represented in different colors for each year. A phylogenic tree of HA genes (a) and antigenic distance of H9N2 isolated strains to the vaccine strain F98 (b and c) are shown. The *y* axis represents the antigenic distance of the selected H9N2 isolates to the vaccine strain (F98), which is natural number rather than log_10_ or log_2_. The distance of the F98 strain to its own antisera was 0. The *x* axis shows the isolation year. The antigenic distance is calculated by Disij = $\sqrt{\overset{L}{\underset{\text{l}=1}{\sum }}{\left(v_{\text{il}}-a_{\text{jl}}\right)}^{2}}$, where *L* is the dimension of the HI matrix; 1 unit distance is 1 HI titer; MDS, multidimensional scaling.

To analyze the genetic evolution of the HA gene of the H9N2 isolates, 113 representative isolates were selected to perform HA gene sequencing and further analysis based on geographical and host differences. These included 21 viruses isolated in 2013, 42 in 2014, 23 in 2015, 7 in 2016, 11 in 2017, and 9 in 2018. Online HA sequences were used for the phylogenetic tree, including 4 in 2013, 3 in 2014, 7 in 2015, 11 in 2016, 8 in 2017, and 9 in 2018. Phylogenetic analysis showed that the HA genes of the isolates from 2013 to 2018 were all distributed in subgroup II or subgroup III, except for A/chicken/Guangdong/D2703/2013 and A/chicken/Fujian/F1132/2015 and the commercial vaccine strain A/chicken/Shanghai/F/1998 (F/98), which were colocated in subgroup I (Fig. 2a). Among them, the virus strains located in subgroup II included five isolates (5/21, 23.8%) in 2013, 14 isolates (14/42, 33.3%) in 2014, 4 isolates (4/23, 17.4%) in 2015, 1 isolate (1/7, 14.3%) in 2016, and 4 isolates (4/11, 36.4%) in 2017. The subgroup II lineage was represented by A/chicken/Zhejiang/HJ/2007, belonging to the G57 genotype described previously (26).

In addition, a new branch of evolution, subgroup III, began to appear after 2013, including 14 viruses (14/21, 66.7%) isolated in 2013, 28 (28/42, 66.7%) in 2014, 19 (18/23, 78.3%) in 2015, 6 (6/7, 85.7%) in 2016, 7 (7/11, 63.6%) in 2017, and 9 (9/9, 100.0%) in 2018.

To estimate the time when subgroup I, subgroup II, and subgroup III viruses started to appear, the most recent common ancestor (tMRCA) for these time points was estimated based on the HA genes of all H9N2 isolates in the time-scaled phylogenic tree (Fig. S1 in the supplemental material). Subgroup I lineage viruses first emerged in August 1992, with a 95% highest posterior density (HPD) interval from September 1991 to May 1993. The tMRCA of subgroup II viruses was September 1996 (July 1995 to November 1996). The most recently isolated H9N2 strains from 2018 were in subgroup III, of which the tMRCA was estimated as September 2010 (95% HPD interval of January 2010 to May 2011).

### A new antigenic cluster of H9N2 emerged.

To monitor the antigenic map of the H9N2 isolated viruses, 27 representative isolates were selected for crossing hemagglutination inhibition (HI) tests, including three viruses isolated in 2013, two in 2014, two in 2015, seven in 2016, seven in 2017, and six in 2018 (Fig. 2a). A vaccine strain F/98 (27) and A/chicken/Shanghai/441/2009 (28) were used as representative viruses of antigenic cluster 1 and antigenic cluster 2, respectively. Four other viruses (A/chicken/Hunan/2106/2009, A/chicken/Jiangsu/A2093/2011, A/chicken/Shanghai/B469/2011, and A/chicken/Hunan/B445/2011) were used in this assay. The antigenic distances of the selected H9N2 strains are shown in Table S3. The antigenic distances of selected H9N2 strains to vaccine strain (F98) were visualized in Fig. 2b. Partial H9N2 isolates from 2013 to 2017 gathered in antigenic cluster 2, including one (1/3, 33.3%) virus isolated in 2013, one (1/2, 50%) in 2015, one (1/7, 14.3%) in 2016, and three (3/7, 42.9%) in 2017 (Fig. 2b, c). A new antigenic cluster (cluster 3) started to appear after 2013, consisting of two (2/3, 66.7%) viruses isolated in 2013, two (2/2, 100.0%) in 2014, one (1/2, 50.0%) in 2015, six (6/7, 85.7%) in 2016, and four (4/7, 57.1%) in 2017. Moreover, all six (100%) H9N2 AIVs isolated in 2018 belonged to antigenic cluster 3.

### Viruses from antigenic cluster 3 presented a faster substitution rate.

**FIG 3:**
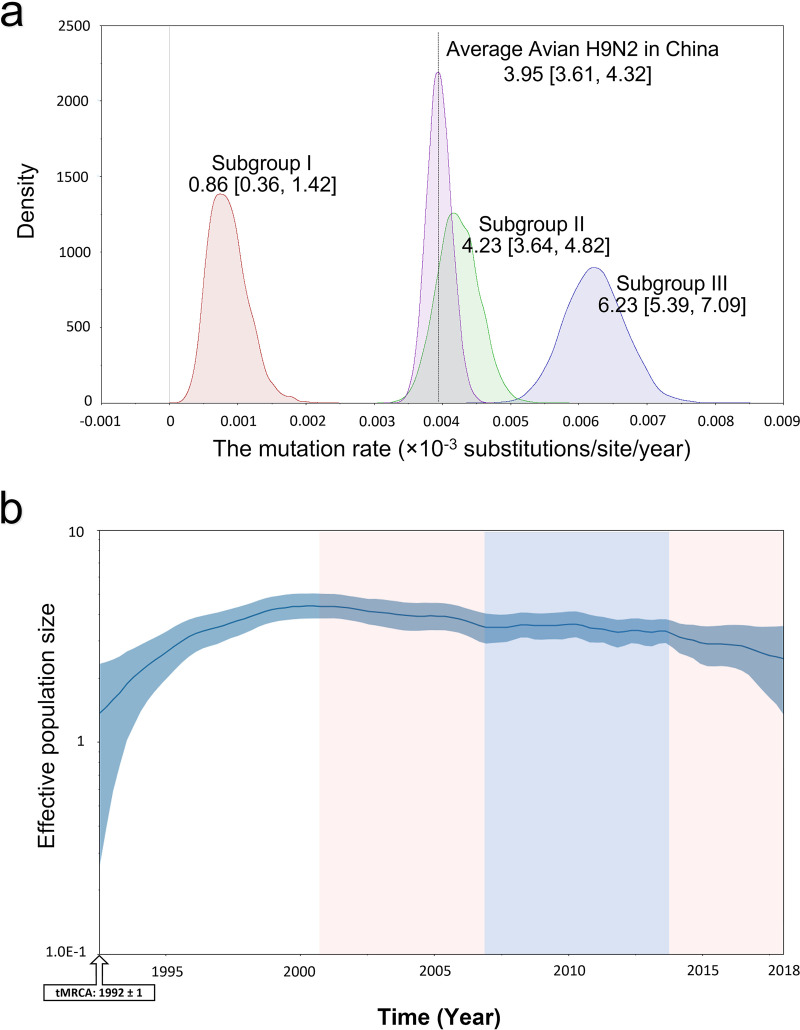
Substitution rate and diversity of H9N2 HA estimated in BEAST. (a) The substitution rate (substitutions/site/year) of the HA ORF of the main clades of H9N2. All 126 lab-isolated H9N2 strains were analyzed. The values show the mean rate with a 95% HPD interval. (b) Bayesian skyride plot (BSP). The *y* axis shows the effective population size (log value). The marginal density of the mean rate data estimated by Bayesian skyride are displayed by Kernel density estimation (KDE) in tracer.

To study evolutionary rates of the HA gene for H9N2 viruses over time, each antigenic cluster virus was analyzed by calculating the substitution rate of HA genes. The average evolution rate of the HA gene of H9N2 AIVs from 1994 to 2018 was estimated, with 3.95 × 10^−3^ (95% HPD interval of 3.61E−3 to 4.32E−3) substitutions/site/year over a 25-year period (Fig. 3a). The HA gene of antigenic cluster 1 viruses (subgroup I) had a slower evolution rate (0.86 × 10^−3^ substitutions/site/year; 95% HPD interval of 0.36E−3 to 1.42E−3), with only 18.75% of the average evolution rate of the HA gene of H9N2 AIVs. In contrast, the HA genes of antigenic cluster 3 viruses (subgroup III) evolved most rapidly with 6.23 × 10^−3^ substitutions/site/year, 1.5-fold faster than that of the average substitution rate of HA genes for H9N2 viruses (Fig. 3a).

To investigate whether this evolutionary rate of HA genes influenced its gene diversity, the HA gene diversity of H9N2 viruses was visualized in the Bayesian skyride plot (BSP) of the viral effective population size (Fig. 3b). The population size of H9N2 HA reached a peak in 2001. From 2001 to 2013 the population sizes were stable. However, after 2013, the population size decreased rapidly. The population has been declining since 2013. At the same time, the subgroup III virus emerged and coexisted with subgroup II viruses, whose evolutionary rates of HA genes were faster than subgroup I viruses. Population size is another measure of genetic diversity. Thus, it seemed that the high evolution rate of the HA gene may lessen genetic diversity.

### Potential antigenic-related amino acid residues.

**FIG 4:**
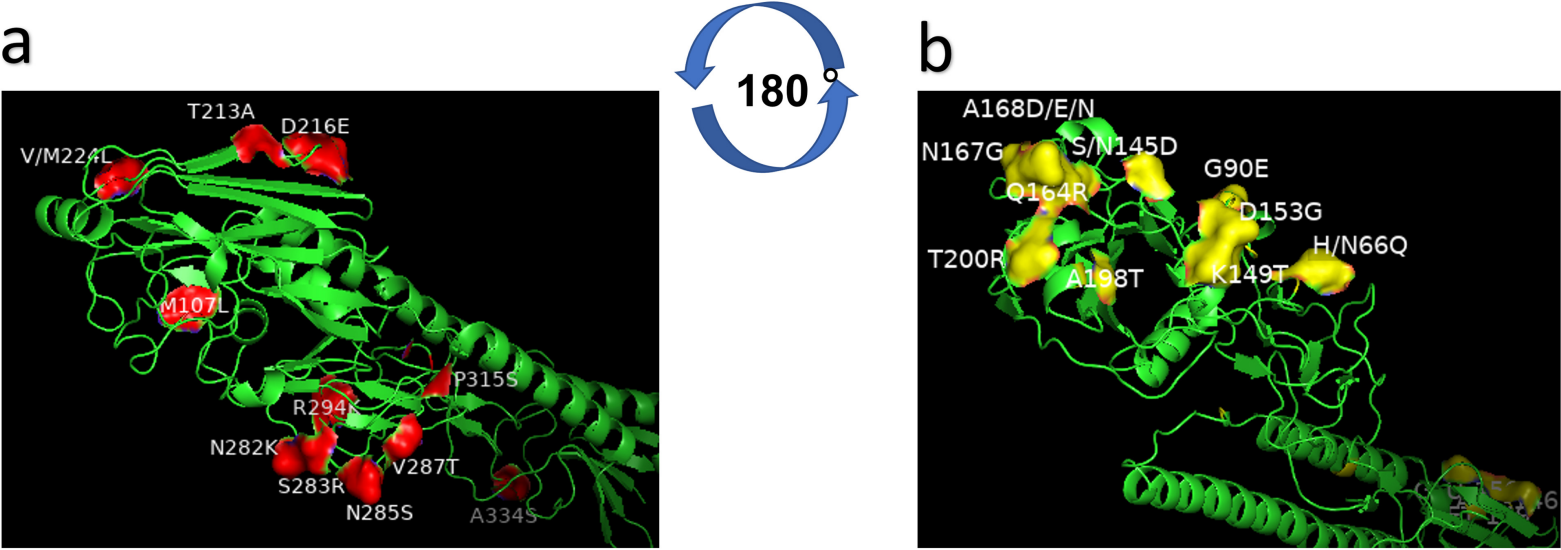
The mutated amino acid residues on the HA1 protein (H9 protein structure). The model was built based on the template A/swine/Hong Kong/9/98, and the modeled strain was A/chicken/Fuzhou/I69/2018. (a) Potential residues in HA contributing to the antigen change between vaccine strains (subgroup I) and circulating strains (subgroups II and III). Residues (in red) (M96L, T205A, D208E, Q226L, N273K, S274R, N275S, V276T, R285K, P306S, and A325S in H3 numbering) are different from the vaccine group (cluster 1) to cluster 2 and 3 (circulating strains in subgroup II and III). (b) Potential residues in HA contributing to the antigen change between subgroup II and subgroup III. Residues (in yellow) (N/H57Q, G81E, S/N133D, K137T, D145G, Q156G, N159G, A160D/N/E, V/A190T, and T192R in H3 numbering) differ between cluster 2 and cluster 3.

The homology of HA protein from different antigenic clusters was analyzed to assess the potential amino acid residues resulting in antigen drift. Eleven amino acid residues were conserved in vaccine cluster viruses (4 classical vaccine strains used in China) but mutated into the same amino acid residues both in cluster 2 and 3 viruses, including M96L, T205A, D208E, Q226L, N273K, S274R, N275S, V276T, R285K, P306S, and A325S (H3 numbering), indicating that these residues in HA may contribute to the antigen change between cluster 1 and clusters 2/3 (Table 2; Fig. 4a).

**TABLE 2:** Specific amino acid mutations on HA of the representative H9N2 isolated strains

Subgroup	Potential antigenic sites																													RBS^*a*^		
	H3 numbering	57	81	96	133	135	137	145	156	158	159	160	183	188	189	190	192	193	199	205	208	216	273	274	275	276	285	306	325	226	227	228
	H9 numbering^*b*^	66^*c*^	90^*c*^	107^*d*^	145^*c*^	147	149^*c*^	153^*c*^	164^*c*^	166	167^*c*^	168^*c*^	191	196	197	198^*c*^	200^*c*^	201	207	213^*d*^	216^*d*^	224^*d*^	282^*d*^	283^*d*^	285^*d*^	287^*d*^	294^*d*^	315^*d*^	334^*d*^	234	235	236
	HA1 residues^*e*^	48	72	89	127	129	131	135	146	148	149	150	173	178	179	180	182	183	189	195	198	206	264	265	267	269	276	297	316	216	217	218
Cluster 1	NJ/99-vac	H	G	M	N	T	K	D	Q	S	N	A	N	D	T	T	T	N	D	T	D	M	N	S	N	V	R	P	A	Q	Q	G
	SD/96-vac	H	G	M	S	T	K	D	Q	D	N	A	N	D	T	A	T	N	D	T	D	V	N	S	N	V	R	P	A	Q	Q	G
	GD/94-vac	H	G	M	S	T	K	D	Q	S	N	A	N	D	T	A	T	N	D	T	D	V	N	S	N	V	R	P	A	Q	Q	G
	F/98-vac	H	G	M	S	T	K	D	Q	N	N	A	N	D	T	A	T	N	D	T	D	M	N	S	N	V	R	P	A	Q	Q	G
Cluster 2	A2093	H	G	L	S	T	K	D	Q	N	N	A	N	D	T	V	T	N	D	A	E	L	K	R	S	T	K	S	S	L	Q	G
	441	H	G	L	S	T	K	D	Q	N	N	A	N	D	T	V	T	N	D	A	E	L	K	R	N	T	K	S	S	L	Q	G
	2106	H	G	L	S	T	K	D	Q	N	N	A	N	E	T	A	T	N	D	A	E	L	K	R	S	T	K	S	S	L	Q	G
	B469	R	E	V	S	T	K	D	Q	N	N	N	N	D	T	A	T	N	D	A	D	L	K	S	N	T	R	P	A	L	Q	G
	B445	H	G	L	S	T	K	D	Q	N	N	A	N	D	T	A	T	N	D	A	E	L	K	R	S	T	K	S	S	L	Q	G
	F1650	H	G	L	S	T	K	D	Q	N	N	A	N	E	T	V	T	N	D	A	E	L	K	R	S	T	K	S	S	L	M	G
	G755	N	G	L	S	T	K	D	Q	N	N	A	N	E	T	V	T	N	D	A	E	L	K	R	S	T	K	S	S	L	M	G
	H97	N	G	L	S	T	K	D	Q	N	N	A	N	E	T	A	T	N	D	A	E	L	K	R	S	T	K	S	S	L	M	G
	H1258	H	G	L	S	T	K	D	Q	N	N	A	N	D	T	V	T	N	D	A	E	L	K	R	S	T	K	S	S	L	M	G
	H1301	H	G	L	S	T	K	D	Q	N	N	A	N	D	T	A	T	N	D	A	E	L	K	R	S	T	K	S	S	L	M	G
	D1021	H	G	L	S	T	K	D	Q	D	N	A	N	D	T	V	T	N	D	A	E	L	K	R	S	T	K	S	S	L	M	G
Cluster 3	D2641	Q	E	L	N	T	T	G	R	N	G	D	N	D	T	T	R	D	D	A	E	L	K	R	S	T	K	S	S	L	M	G
	D345	Q	E	L	D	T	T	G	R	N	G	D	N	D	T	T	R	N	D	A	E	L	K	R	S	T	K	S	S	L	M	G
	E2290	Q	E	L	D	T	T	G	R	N	G	N	N	D	T	T	R	D	D	A	E	L	K	R	S	T	K	S	S	L	M	G
	E552	Q	E	L	D	T	T	G	R	N	G	D	N	D	D	T	R	N	D	A	E	L	K	R	S	T	K	S	S	L	M	G
	F2170	Q	E	L	D	T	T	G	R	N	G	D	N	D	T	T	R	N	D	A	E	L	K	R	S	T	K	S	S	L	M	G
	G378	Q	E	L	D	T	T	G	R	N	G	E	N	D	D	T	R	G	D	A	E	L	K	R	S	T	K	S	S	L	M	G
	G705	Q	E	L	D	T	T	G	R	N	G	E	N	D	T	T	R	D	D	A	E	L	K	R	S	T	K	S	S	L	M	G
	G1451	Q	E	L	D	T	T	G	R	N	G	N	N	D	D	T	R	N	D	A	E	L	K	R	S	T	K	S	S	L	M	G
	G1586	Q	E	L	D	T	T	G	R	N	G	E	N	D	V	T	R	G	D	A	E	L	K	R	S	T	K	S	S	L	M	G
	G1728	Q	E	L	D	T	T	G	R	D	G	N	N	D	D	T	R	N	D	A	E	L	K	R	S	T	K	S	S	L	M	G
	G1921	Q	E	L	D	T	T	G	R	D	G	N	N	D	T	T	R	G	D	A	E	L	K	R	S	T	K	S	S	L	M	G
	H224	Q	E	L	D	T	T	G	R	D	G	N	N	D	D	T	R	N	D	A	E	L	K	R	S	T	K	S	S	L	M	G
	H514	Q	E	L	D	T	T	G	R	D	G	N	N	D	D	T	R	S	D	A	E	L	K	R	S	T	K	S	S	L	M	G
	H1114	Q	E	L	D	T	T	G	R	N	G	E	N	D	D	T	R	G	D	A	E	L	K	R	S	T	K	S	S	L	M	G
	H1517	Q	E	L	D	T	T	G	R	N	G	E	N	D	D	T	R	G	D	A	E	L	K	R	S	T	K	S	S	L	M	G
	I25	Q	E	L	D	T	N	G	R	D	G	N	N	D	T	T	R	N	D	A	E	L	K	R	S	T	K	S	S	L	M	G
	I69	Q	E	L	D	T	N	G	R	N	G	N	N	D	D	T	R	N	D	A	E	L	K	R	S	T	K	S	S	L	M	G
	I84	Q	E	L	D	T	T	G	R	N	G	E	N	D	E	T	R	R	D	A	E	L	K	R	S	T	K	S	S	L	M	G
	I97	Q	E	L	D	T	T	G	R	N	G	E	N	D	D	T	R	G	D	A	E	L	K	R	S	T	K	S	S	L	M	G
	I112	Q	E	L	D	T	T	G	R	N	G	E	N	D	D	T	R	G	D	A	E	L	K	R	S	T	K	S	S	L	M	G
	I256	Q	E	L	D	T	T	G	R	N	G	E	N	D	D	T	R	G	D	A	E	L	K	R	S	T	K	S	S	L	M	G

a RBS, receptor binding site.

b The whole length of amino acid sequence of HA protein.

c Key locations identified in this research where mutations contribute to the antigenic distance between clusters 1 and 2.

d Locations holding mutations that contribute to the antigenic distance between vaccine strains and isolates and potential location under selection from vaccines.

e The HA1 protein sequence without 10 resudies at the beginning of HA protein.

Investigation of the key amino acids characterizing antigenic properties of strains in cluster 2 showed 10 mutated amino acid residues in the HA protein between cluster 2 viruses and cluster 3 viruses, including N/H57Q, G81E, S/N133D, K137T, D145G, Q156G, N159G, A160D/N/E, V/A190T, and T192R (Table 2; Fig. 4b). In a subsequent analysis, G81E, 137K, 145D, 156Q, 159N, A160D/N/E, and T192R were conserved in both cluster 1 and cluster 2 viruses but mutated in the cluster 3 viruses. These results indicate that these seven amino residues may be important for the antigenic property of cluster 3 viruses and influence the protective efficacy of the inactivated H9N2 AIV vaccine.

### Evolution of the potential antigenic-related amino acid residues.

**FIG 5:**
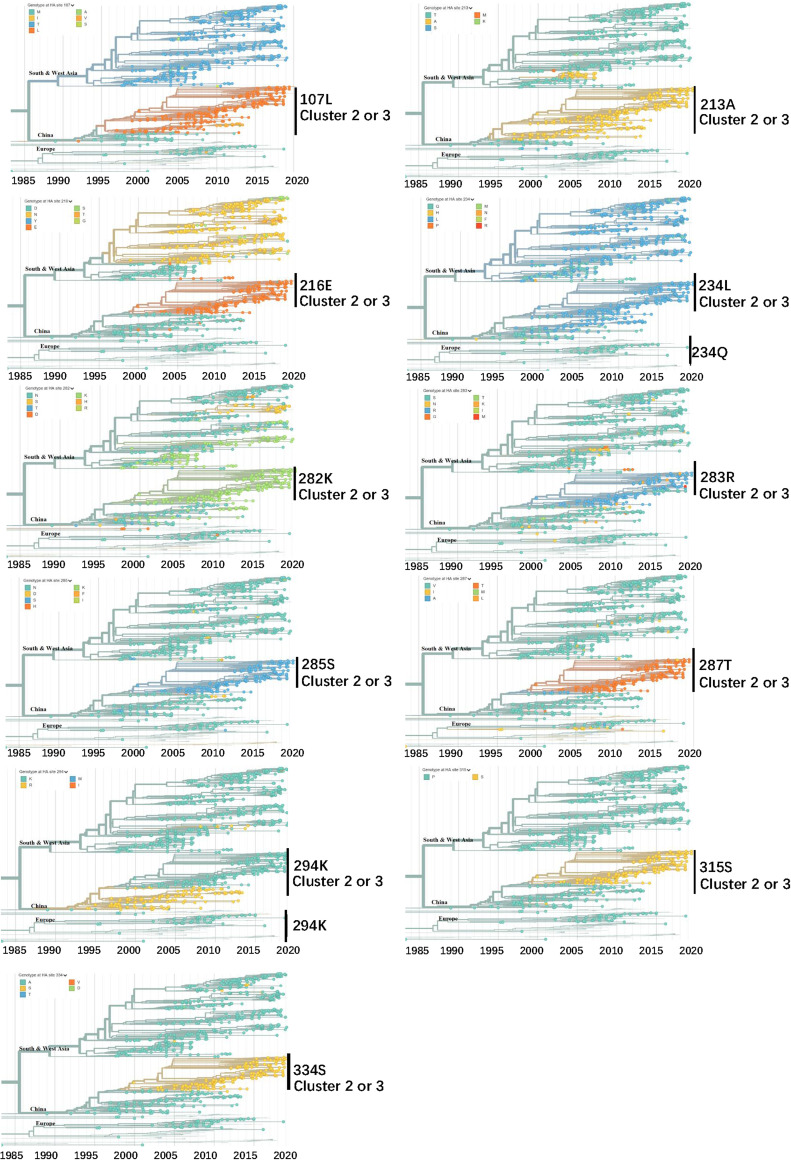
Real-time dynamics of 11 potential antigenic-related amino acid residues comparing vaccine strains (subgroup I) and current isolated strains (subgroups II and III). The locations are numbered following the H9 numbering commencing from the start of the ORF. Phylogenic trees are colored by HA amino acid positions of interest. Legends at the top left display the respective HA sites of the H9N2 strains globally. Regions are marked at the node of clades. Data and the analysis are from Nextstrain (58). Shown are 1,092 of 1,332 genomes sampled between January 1976 and June 2019. The data were filtered to avian (1,270) from Africa (115), China (358), Europe (55), South Asia (126), Southeast Asia (93), and West Asia (395).

**FIG 6:**
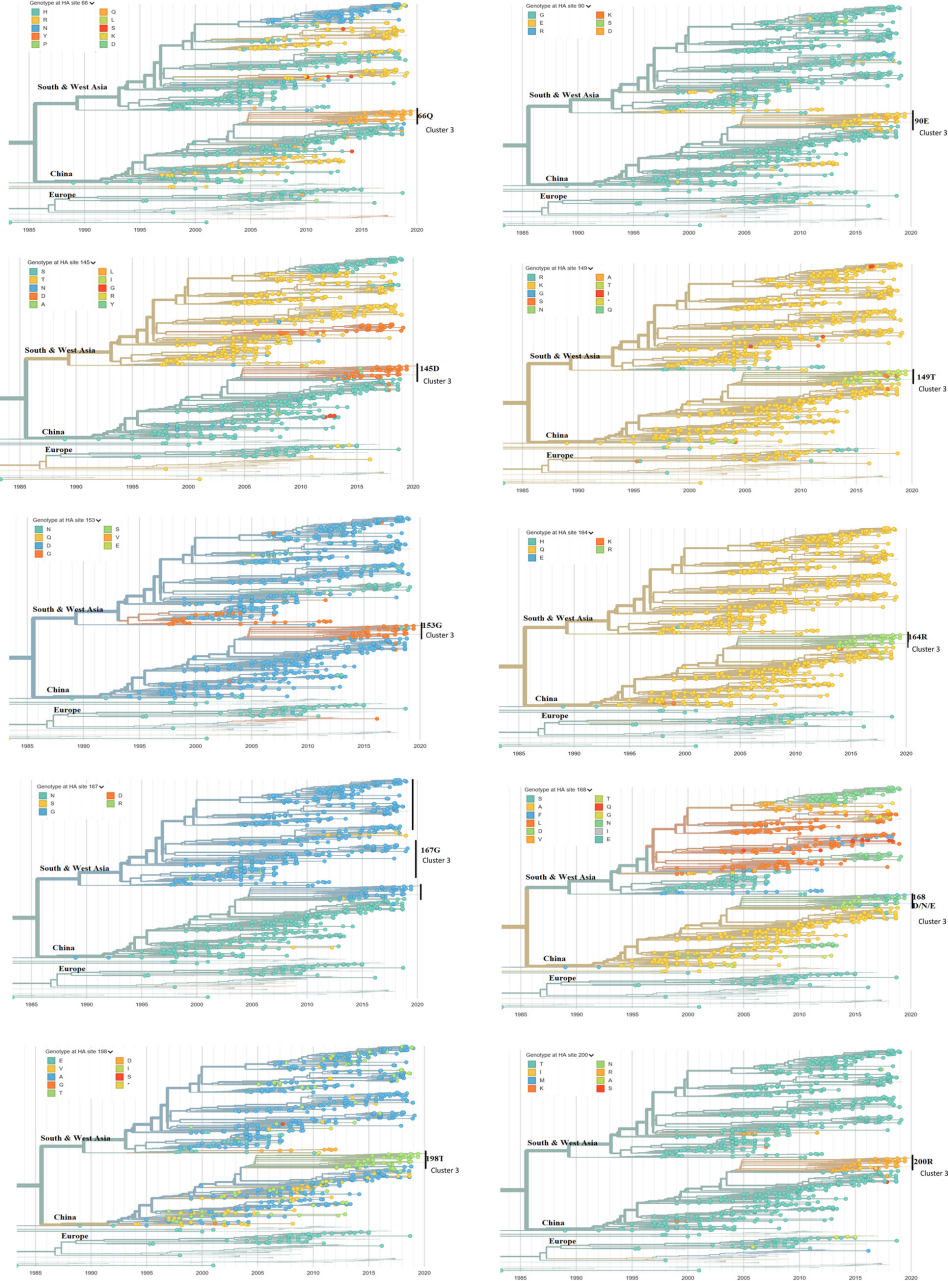
Real-time dynamics of 10 potential antigenic-related amino acid residues comparing cluster 2 (subgroup II) and cluster 3 (subgroup III). The locations are numbered following H9 numbering starting from the start of the ORF. Phylogenic trees are colored by amino acid positions of interest on HA. Legends at the top left display the respective HA sites of the H9N2 strains globally. Regions are marked at the node of clades. Data and the analysis are from Nextstrain (58). Shown are 1,092 of 1,332 genomes sampled between January 1976 and June 2019. The data were filtered to avian (1,270) from Africa (115), China (358), Europe (55), South Asia (126), Southeast Asia (93), and West Asia (395).

To gain information on the evolution of potential antigenic amino acid residues on HA from H9N2 over time in nature, we analyzed HA proteins of H9N2 isolates and other viruses submitted to NCBI. Of the 11 potential antigenic amino acids characteristic of cluster 1, 10 were dominant patterns in China, including M96L, T205A, D208E, Q226L, N273K, N275R, V276T, R285K, P306S, and A325S in HA protein (H3 numbering). Currently, S274R is the predominant amino acid for H9N2 HA, mixed with some S274 (Fig. 5). Moreover, all mutations contributing to the antigenic change from cluster 1 to cluster 2 were equally distributed for the natural H9N2 AIVs, including N/H57Q, G81E, S/N133D, K137T, D145G, Q156G, N159G, A160D/N/E, V/A190T, and T192R (Fig. 6).

## DISCUSSION

The H9N2 virus is a predominant subtype of AIV circulating in Asia and other countries all over the world (29–33). H9N2 viruses not only cause large economic losses in poultry but also provide internal genes for novel subtype influenza viruses, such as H7N9, H10N8, and H5N1, that threaten human health (26, 34). Live poultry markets (LPMs) are considered key public place of the transmission and circulation of influenza viruses in poultry or from poultry to humans (35–37).

Our surveys of LPMs conducted in China from 2013 to 2018 demonstrate that H9N2 AIVs are still prevalent, as described previously (28). An increasing trend of annual isolation rate was observed in poultry markets from 2013 to 2015 and reduced from 2015 to 2018. Of note, the total sampling number in surveillance fluctuated annually. Small sampling numbers (samples from Shanghai in 2015 and samples from Fujian; Table 1) and a biased sampling (e.g., samples from Shandong in 2015 and samples from Shanghai in 2016 were from sick poultry; Table 1) increased the margin of error in the estimation of annual isolation rate of H9N2 in Southern China. In a previous survey, a fluctuating annual isolation rate of H9N2 avian influenza virus was observed in 2013 (14.2%) and 2014 (11.9%) compared to in 2009 (15.18%) and 2012 (8.20%) (28). The common reduced annual isolation rate of H9N2 AIV in China detected in surveillance was also influenced by national or local policy on LPMs. The sale of live poultry in LPMs was prohibited in China in 2013 and 2014 due to the outbreak of H7N9 in humans in 2013 (38). This prohibition largely reduced the isolation rate of all AIVs in LPMs in Southern China. However, the H9N2 AIV was still the predominant subtype LPAIV circulating in poultry in Southern China. Based on our analysis, the overall isolation rate of H9N2 was about 70% in total AIV-positive samples in Southern China between 2013 and 2018. Together with the continuous outbreaks in domestic poultry in other countries (4, 39, 40), follow-up monitoring and control of H9N2 AIV are necessary even after prolonged marketing prohibitions and vaccination programs.

Together with the high isolation rate and long-term endemic circulation, H9N2 AIVs in mainland China are undergoing a rapid antigenic drift. The hypothesis that vaccination triggered rapid evolution leading to antigenic drift was reported in Korea only 4 years after the national vaccination program was applied in poultry in 2007 (22). Similarly, this antigenic drift on H9 HA was identified from H9N2 viruses isolated from chickens and reported in many studies (41, 42). These studies called for caution regarding immunization failure resulting from antigenic drift and explained the prevalence of H9N2 AIV. However, it remained unclear how and how rapidly the antigenic changes on the HA gene of H9N2 strains from the market occurred. To analyze this, we combined phylogenetic and antigenic analyses based on the current genetic data.

Phylogenic analysis of 113 H9N2 strains isolated from LPMs in Southern China from 2013 to 2018 provided sufficient circulating and evolutionary information of H9N2 in local poultry. Consistent with previous studies indicating G9 (A/chicken/Hong Kong/G9/1997) lineage (h9.4.2) (43) as the dominant H9N2 in mainland China, the latest isolates were also G9 lineage but far from the original representative strains BJ94 (A/chicken/Beijing/1/1994) and Y280 (A/duck/Hong Kong/Y280/97) and the vaccine strains in the 1990s (14, 44). In addition, the most recent isolated strains antigenically evolved into a new group from 2013. Phylogenetic analysis showed that the estimated time for the emergence of the new antigenic cluster was between 2010 and 2011. This bias may be due to the lack of precise time data from isolated strains and the small sampling of virus strains selected in the antigenic assay. However, a rapid and clear subdivision of the isolated H9N2 strains (belonging to subgroup II in current G9 lineage) matched the two new antigenic groupings. This guaranteed the confidence interval when we estimated the substitution rate of H9N2 strains in a new antigenic group (cluster 2). Without considering the antigenic difference, the average substitution rate of the HA gene from H9N2 AIV in poultry in China was estimated as 3.95 × 10^−3^ substitutions/site/year. This substitution rate increased almost 1.5-fold (6.23 × 10^−3^ substitutions/site/year) when antigenic differences were involved.

Of note is that this is the first time the substitution rate of H9 HA (open reading frame [ORF] region) using the sequences from the influenza database has been estimated. These rates are similar to the estimations using the same method for H3 (HA1 domain) in humans (5.7 × 10^−3^ substitutions/site/year) (45). For better comparison, the mean H9 substitution rate (HA1 domain) of antigenic cluster 2 was 7.43 × 10^−3^ substitutions/site/year, still slightly higher than the H3 viruses circulating in humans. This indicated that H9N2 AIV underwent changes in evolution rate, with rates faster than those from human seasonal strains. This may be due to inefficiencies in vaccination and high living density of chickens that contributed to more transmission into new hosts. However, more research is needed to better understand the reasons triggering the substitution rate in addition to selection pressure resulting from vaccination.

We found 21 amino acid mutations on H9 antigenic sites (in region 66 to 334 on the H9 protein sequence) specific to strains in antigenic cluster 2, different from those in cluster 1 as well as three vaccine strains. Referring to the global online database of AIV H9N2 in Nextstrain, all these mutations evolved after 2013 and became dominant in Southern China. This explains why the isolation ratios in LPMs from 2013 to 2018 did not decrease even though vaccines had been used long term in poultry, with reports that the H9N2 virus continues to circulate in vaccinated chickens (26, 46).

Structural analyses of the HA1 protein to predict key potential antigenic sites dominating the subgroup of current H9N2 isolates from field markets in Southern China were performed. The four surface locations (145, 168, 198, and 235) at the top of the HA head also show high entropy in real-time tracing (47). This indicates high diversity of these four locations. Overall, mutations at these potential antigenic sites occurred in China (mainly) and in Southern Asia, whereas in China, new genotypes G57 showed the tendency to become dominant. In detail, D145 started to appear in China and South Asia in as early as 2003. Mutations at 198 and 235 emerged and became dominant in China after 2012. To date, location 168 was more diverse with mutations from alanine to other amino acids after 2005. The L226 site (234 in H9 numbering), identified as an important host-receptor binding site (48), had been the stable dominant form on HA of the H9N2 AIV circulating in China. However, at location 235, we identified that the change from glutamine to methionine occurred in 2013, which was consistent with the online data of the H9N2 AIV all over China; however, Q235 was the stable dominant form in the European area where no H9N2 vaccine had been applied. The amino acids of the surface locations were conserved in Europe but became dominant in China after 2013. However, there may be data bias given that many European countries did not monitor LPAI H9N2 in poultry.

In summary, our study shows that the currently circulating H9N2 viruses form a new antigenic group to those from vaccine strains and are also clearly divided into new subgroups in phylogenic trees. The declining diversity together with the high substitution rate of H9N2 strains in the new antigenic group suggest that vaccination triggers the evolution of H9N2 into new antigenic groups. Our study provides a baseline reference for evolutionary studies of H9N2 viruses and vaccination strategies in poultry.

## MATERIALS AND METHODS

### Ethics statement.

All animal experiments were conducted in accordance with the recommendations of the Guide for the Care and Use of Laboratory Animals of the Ministry of Science and Technology of the People’s Republic of China and the Netherlands. The protocols (SHVRI-SZ-20190730-01 and SHVRI-SZ-20190906-02) used in the study were approved by the Animal Care and Use Committee of Shanghai Veterinary Research Institute (SHVRI).

### Sample collection and virus isolation.

Between 2013 and 2018, a total of 13,981 oropharyngeal swabs and cloaca swabs were collected from chickens, ducks, geese, and pigeons in local markets from nine provinces in China (Guangzhou, Fujian, Jiangxi, Zhejiang, Shanghai, Anhui, Jiangsu, Shandong, and Hunan). Swab samples were inoculated in the allantoic cavity of 10-day-old specific-pathogen-free (SPF) chicken egg embryos. The harvested allantoic fluids were tested by hemagglutination assay. Total viral RNAs of HA-positive allantoic fluids were extracted to detect influenza viruses by amplifying the M gene by PCR. The primers used for this detection were 5′-TTCTAACCGAGGTCGAAAC-3′ (M-229U) and 5′-AAGCGTCTACGCTGCAGTCC-3′ (M-229L). Hemagglutination inhibition (HI) assays were then used as antiserums specific for H9 subtype influenza viruses.

### Phylogenic analysis, substitution rate, and gene diversity of the HA gene.

The viral RNA was extracted, and reverse transcription was performed with the Uni12 primer as previously described (49, 50). Complete HA sequences of H9N2 isolated strains were edited using EditSeq in Lasergene 7.0. The full-length nucleotide sequences of HA genes of H9N2 strains collected from 2013 to 2018 in China were submitted to the Global Initiative on Sharing Avian Influenza Data (GISAID) and GenBank database (Table S1 in the supplemental material). Based on previous research (28), representative strains of H9N2 in China and from other Asian and American countries were selected for a neighbor-joining (NJ) tree using MEGAx. The TN93 (Tamura-Nei, 93) +G (0.39) model was used with 1,000 bootstraps.

To generate a time scale tree of the whole lineage containing the H9N2 strains isolated from this lab (51), we included all G9 lineage strains from the Yang et al. data set (14) plus a few additional representatives for the lineage indications from mainland China. The exact date information was adjusted. Before running the time-measured phylogenetic analysis, the clock-likeness of the HA sequence data set was analyzed with TempEst (52). Six outliers (FJ190132, EPI_ISL_260531, AF156378, CY005632, EPI_ISL_146723, and KF188265) were identified and removed from the data set. We then constructed time-scaled phylogenies using a Bayesian Markov chain Monte Carlo (MCMC) framework implemented in BEAST (v1.10.2) (53). Analysis was conducted by using the SRD06 nucleotide substitution model, the Bayesian Skyride coalescent model, and an uncorrelated log normal relaxed molecular clock. Four independent MCMC runs of 1 × 10^8^ states sampling each 1 × 10^4^ steps were performed to obtain an effective sample size of >200. Maximum clade credibility trees were reconstructed with 10% burn-in, and the posterior distribution of relevant parameters was assessed in FigTree version 1.4.4. The Skyride plot was generated in Tracer with an estimation for the mean substitution rate (substitutions/site/year).

### Antigenic assay.

According to the phylogenetic tree of the HA gene, 27 H9N2 viruses were selected from the isolated strains from 2013 to 2018; six stored virus strains from previous research (A/chicken/Hunan/B445/2011, A/chicken/Shanghai/B469/2011, A/chicken/Jiangsu/A2093/2011, A/chicken/Shanghai/441/2009, A/chicken/Hunan/2106/2009, and A/chicken/Shanghai/F/1998) were selected for the antigenic analysis. Briefly, specific antisera with inactivated virus isolates were produced in SPF Leghorn chickens with adjuvant Montanide ISA 71VG (SEPPIC). Four-week-old SPF Leghorn chickens were vaccinated with a total of 1.5 mL by intramuscular injection. After 3 weeks, sera were collected from vaccinated chickens. The hemagglutination inhibition (HI) assay was conducted to test cross-reactivity between virus and antisera as described previously by the World Organization for Animal Health (OIE) (54). First, the sera of each virus strain were diluted 2-fold with phosphate-buffered saline (PBS) on a v-type microreaction plate. Then, allantoic fluid diluted to 4 U antigen per 25 μL was added to each well. After 30 min at room temperature, 25 μL of 0.5% red blood cells was added to each well. The results were determined after a 25- to 30-min reaction. Each HI value was calculated (Table S2). This HI assay data set was used to calculate antigenic distances between the different H9N2 isolates by using the method described previously (55) in R studio (Data S1). A scatter plot of all the H9N2 isolates with their distances to the vaccine strain (F98) (Data S2) was programmed in R studio (Data S1).

### Molecular evolution analysis.

To investigate the amino acid mutations that played an important role in the appearance of a new antigenic cluster, the HA protein sequences of selected 2013 to 2018 H9N2 isolated viruses and six previous H9N2 viruses were aligned and compared. Based on the new clustering in the antigenic assay, we searched for specific molecular changes on the amino acid sequences that were different between the antigenic clusters. To achieve the three-dimensional (3D) position of the identified molecular changes on the sites of the HA protein, the HA1 structure of H9 was simulated. A/swine/Hong Kong/9/98 (H9N2) (1JSD) (56) was used as the template with a sequence identity of 86.21. The HA structure model was built with 0.96GMQE (global model quality estimation) and 0.23QMEAN (57).

We then studied the real-time dynamic changing of these potential antigenic sites by generating a phylogenetic tree of the HA protein at every site on one practical online database (https://nextstrain.org) (58). To zoom in on the evolutionary situation in China, we filtered the H9N2 sequence data from avian influenza in China from 1968 to 2020. We selected and colored the interesting potential antigenic sites on the HA protein so that the distribution and diversity of the amino acids at that site would display in a time scale tree. Thus, the molecular evolution of one amino acid site located on the HA protein was traced based on real-time H9N2 data all over China.

### Data availability.

All the nucleotide sequences of HA and NA genes from H9N2 isolated in Southern China from 2013 to 2018 were submitted in the GISAID database (https://platform.gisaid.org/epi3/cfrontend#80368) with isolate IDs from EPI_ISL_462482 to EPI_ISL_462520. These HA sequences were submitted to the GenBank database (OK563122 to OK563235).

The G9 lineage strains recovered from the data set in reference 14 are available in the GenBank Influenza Virus Database (http://www.ncbi.nlm.nih.gov/genomes/FLU/FLU.html) hosted by NCBI. Details are at https://doi.org/10.1371/journal.pcbi.1007189.s012 (14).

Data and analysis from Nextstrain (https://nextstrain.org/flu/avian/h9n2/ha) consisted of 1,092 of 1,332 genomes sampled between January 1976 and June 2019. The data used in this research were filtered to avian (1,270) from Africa (115), China (358), Europe (55), South Asia (126), Southeast Asia (93), and West Asia (395).
