# Supplementary material for: Molecular and Antigenic Characterization of Avian H9N2 Viruses in Southern China
Source: Microbiol Spectr. 2022 Jan 12;10(1):e00822-21. doi: 10.1128/spectrum.00822-21 (PMC8754122; doi:10.1128/spectrum.00822-21)
Supplement: SUPPLEMENTAL FILE 1 — Supplemental material. Download SPECTRUM00822-21_Supp_1_seq11.pdf, PDF file, 0.7 MB [file spectrum00822-21_supp_1_seq11.pdf]

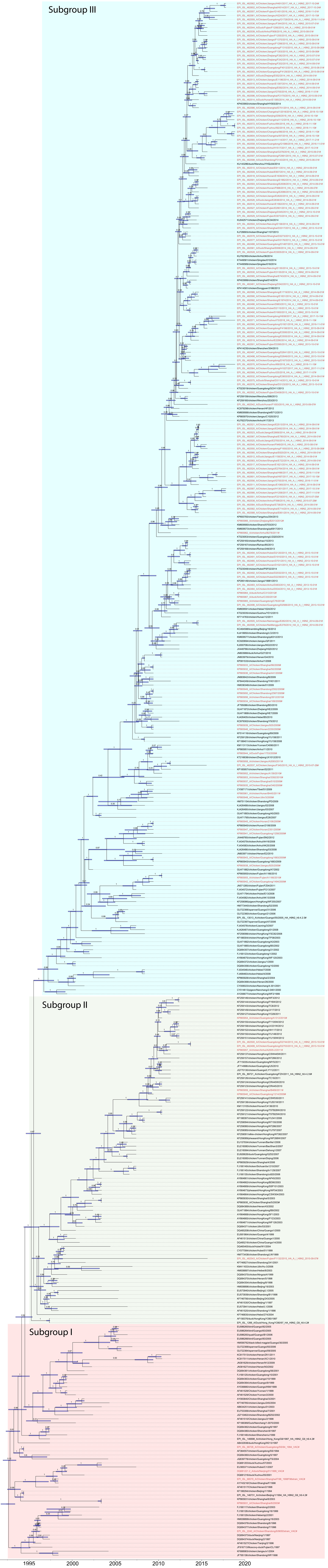
Subgroup III
Subgroup II
Subgroup I
1995
2000
2005
2010
2015
2020

**Supplementary File1. Time-scale tree of HA gene of the G9 lineage of H9N2.** The lab-isolated H9N2 strains from the 2013 to 2018 surveillance are marked with "EPI_ISL_". The G9 lineage strains recovered from the data set of Yang et al 2019 (1) are given in black.

## Reference


1. Yang J, Müller NF, Bouckaert R, Xu B, Drummond AJ. 2019. Bayesian phylodynamics of avian influenza A virus H9N2 in Asia with time-dependent predictors of migration. PLoS computational biology 15:e1007189.

Table S1. The submission of sequence date in GIASID and GeneBank.

| Isolate_Name | Country | Province | Host | Collection_Date | Segment_Ids GISAID | Segment_Ids GeneBank |
|---|---|---|---|---|---|---|
| A/Chicken/Zhejiang/D342/2013 | China | Zhejiang | Chicken | 2013-10 | EPI1742690 | OK563213 |
| A/Chicken/Zhejiang/D345/2013 | China | Zhejiang | Chicken | 2013-10 | EPI1742698 | OK563212 |
| A/Chicken/Anhui/D483/2013 | China | Anhui | Chicken | 2013-10 | EPI1742706 | OK563214 |
| A/Chicken/Anhui/D534/2013 | China | Anhui | Chicken | 2013-10 | EPI1742714 | OK563215 |
| A/Chicken/Hunan/D701/2013 | China | Hunan | Chicken | 2013-10 | EPI1742722 | OK563216 |
| A/Chicken/Hunan/D963/2013 | China | Hunan | Chicken | 2013-10 | EPI1742730 | OK563217 |
| A/Chicken/Hunan/D1021/2013 | China | Hunan | Chicken | 2013-10 | EPI1742738 | OK563208 |
| A/Chicken/Guangdong/D1467/2013 | China | Guangdong | Chicken | 2013-10 | EPI1742746 | OK563218 |
| A/Chicken/Guangdong/D1673/2013 | China | Guangdong | Chicken | 2013-10 | EPI1742754 | OK563219 |
| A/Chicken/Guangdong/D2566/2013 | China | Guangdong | Chicken | 2013-10 | EPI1742810 | OK563220 |
| A/Chicken/Guangdong/D2641/2013 | China | Guangdong | Chicken | 2013-10 | EPI1742818 | OK563209 |
| A/Chicken/Guangdong/D2649/2013 | China | Guangdong | Chicken | 2013-10 | EPI1742826 | OK563221 |
| A/Chicken/Guangdong/D2703/2013 | China | Guangdong | Chicken | 2013-10 | EPI1742834 | OK563210 |
| A/Chicken/Guangdong/D2744/2013 | China | Guangdong | Chicken | 2013-10 | EPI1742842 | OK563222 |
| A/Duck/Shanghai/D3114/2013 | China | Shanghai | Duck | 2013-10 | EPI1743402 | OK563223 |
| A/Chicken/Shanghai/D3123/2013 | China | Shanghai | Chicken | 2013-10 | EPI1743410 | OK563224 |
| A/Chicken/Shanghai/D3176/2013 | China | Shanghai | Chicken | 2013-10 | EPI1743418 | OK563225 |
| A/Chicken/Shanghai/D3273/2013 | China | Shanghai | Chicken | 2013-10 | EPI1743426 | OK563211 |
| A/Chicken/Shanghai/D3317/2013 | China | Shanghai | Chicken | 2013-10 | EPI1743434 | OK563232 |
| A/Chicken/Fujian/D3395/2013 | China | Fujian | Chicken | 2013-10 | EPI1742850 | OK563226 |
| A/Chicken/Fujian/D3458/2013 | China | Fujian | Chicken | 2013-10 | EPI1742858 | OK563227 |
| A/Chicken/NanJing/E135/2014 | China | Jiangsu | Chicken | 2014-9 | EPI1742866 | OK563173 |
| A/Chicken/NanJing/E158/2014 | China | Jiangsu | Chicken | 2014-9 | EPI1742874 | OK563174 |
| A/Duck/Zhejiang/E552/2014 | China | Zhejiang | Duck | 2014-9 | EPI1742898 | OK563169 |
| A/Chicken/Zhejiang/E592/2014 | China | Zhejiang | Chicken | 2014-9 | EPI1742906 | OK563175 |
| A/Chicken/Shanghai/E620/2014 | China | Shanghai | Chicken | 2014-9 | EPI1743442 | OK563176 |
| A/Duck/Shanghai/E658/2014 | China | Shanghai | Duck | 2014-9 | EPI1743450 | OK563171 |
| A/Chicken/Shanghai/E714/2014 | China | Shanghai | Chicken | 2014-9 | EPI1743458 | OK563177 |
| A/Chicken/Shanghai/E722/2014 | China | Shanghai | Chicken | 2014-9 | EPI1743466 | OK563178 |
| A/Duck/Shanghai/E738/2014 | China | Shanghai | Duck | 2014-9 | EPI1743474 | OK563179 |
| A/Chicken/Shanghai/E743/2014 | China | Shanghai | Chicken | 2014-9 | EPI1743482 | OK563180 |
| A/Chicken/shanghai/E751/2014 | China | Shanghai | Chicken | 2014-9 | EPI1743490 | OK563181 |
| A/Chicken/Shanghai/E780/2014 | China | Shanghai | Chicken | 2014-9 | EPI1743498 | OK563182 |
| A/Chicken/Shanghai/E851/2014 | China | Shanghai | Chicken | 2014-9 | EPI1743506 | OK563183 |
| A/Chicken/Jiangsu/E1065/2014 | China | Jiangsu | Chicken | 2014-9 | EPI1742930 | OK563184 |
| A/Duck/Jiangsu/E1158/2014 | China | Jiangsu | Duck | 2014-9 | EPI1742938 | OK563185 |
| A/Chicken/Jiangsu/E1196/2014 | China | Jiangsu | Chicken | 2014-9 | EPI1742946 | OK563186 |
| A/Duck/Hunan/E1385/2014 | China | Hunan | Duck | 2014-9 | EPI1742954 | OK563187 |
| A/Chicken/Hunan/E1397/2014 | China | Hunan | Chicken | 2014-9 | EPI1742962 | OK563229 |
| A/Chicken/Hunan/E1562/2014 | China | Hunan | Chicken | 2014-9 | EPI1742970 | OK563188 |
| A/Chicken/Hunan/E1621/2014 | China | Hunan | Chicken | 2014-9 | EPI1742978 | OK563172 |
| A/Chicken/Hunan/E1639/2014 | China | Hunan | Chicken | 2014-9 | EPI1742986 | OK563189 |

Continued Table S1.

| Isolate_Name | Country | Province | Host | Collection_Date | Segment_Ids GISAID | Segment_Ids GeneBank |
|---|---|---|---|---|---|---|
| A/Chicken/Shangdong/E1719/2014 | China | Shangdong | Chicken | 2014-9 | EPI1743514 | OK563190 |
| A/Chicken/Shandong/E1831/2014 | China | Shangdong | Chicken | 2014-9 | EPI1743522 | OK563191 |
| A/Chicken/Shandong/E1962/2014 | China | Shangdong | Chicken | 2014-9 | EPI1743530 | OK563192 |
| A/Chicken/Shandong/E1974/2014 | China | Shangdong | Chicken | 2014-9 | EPI1743538 | OK563193 |
| A/Chicken/Shandong/E2006/2014 | China | Shangdong | Chicken | 2014-9 | EPI1743546 | OK563195 |
| A/Chicken/Shandong/E2086/2014 | China | Shangdong | Chicken | 2014-9 | EPI1743554 | OK563194 |
| A/Chicken/Anhui/E2290/2014 | China | Anhui | Chicken | 2014-9 | EPI1742994 | OK563170 |
| A/Chicken/Jiangxi/E2492/2014 | China | Jiangxi | Chicken | 2014-9 | EPI1743002 | OK563168 |
| A/Chicken/Jiangxi/E2513/2014 | China | Jiangxi | Chicken | 2014-9 | EPI1743010 | OK563196 |
| A/Chicken/Jiangxi/E2620/2014 | China | Jiangxi | Chicken | 2014-9 | EPI1743018 | OK563197 |
| A/Duck/Jiangxi/E2669/2014 | China | Jiangxi | Duck | 2014-9 | EPI1743026 | OK563198 |
| A/Chicken/Jiangxi/E2764/2014 | China | Jiangxi | Chicken | 2014-9 | EPI1743034 | OK563199 |
| A/Duck/Jiangxi/E2783/2014 | China | Jiangxi | Duck | 2014-9 | EPI1743042 | OK563200 |
| A/Duck/Jiangxi/E2838/2014 | China | Jiangxi | Duck | 2014-9 | EPI1743050 | OK563201 |
| A/Chicken/Fujian/E2901/2014 | China | Fujian | Chicken | 2014-9 | EPI1743058 | OK563202 |
| A/Chicken/Fujian/E3005/2014 | China | Fujian | Chicken | 2014-9 | EPI1743066 | OK563203 |
| A/Chicken/Fujian/E3073/2014 | China | Fujian | Chicken | 2014-9 | EPI1743074 | OK563204 |
| A/Chicken/Fujian/E3135/2014 | China | Fujian | Chicken | 2014-9 | EPI1743082 | OK563205 |
| A/Chicken/Guangdong/E3555/2014 | China | Guangdong | Chicken | 2014-9 | EPI1743562 | OK563206 |
| A/Chicken/Guangdong/E3595/2014 | China | Guangdong | Chicken | 2014-9 | EPI1743090 | OK563207 |
| A/Chicken/Guangdong/E3653/2014 | China | Guangdong | Chicken | 2014-9 | EPI1743098 | OK563228 |
| A/Chicken/Zhejiang/F202/2015 | China | Zhejiang | Chicken | 2015-7 | EPI1743106 | OK563234 |
| A/Chicken/Zhejiang/F242/2015 | China | Zhejiang | Chicken | 2015-7 | EPI1743114 | OK563150 |
| A/Chicken/Zhejiang/F282/2015 | China | Zhejiang | Chicken | 2015-7 | EPI1743122 | OK563231 |
| A/Chicken/Jiangsu/F344/2015 | China | Jiangsu | Chicken | 2015-7-28 | EPI1743130 | OK563233 |
| A/Chicken/Jiangsu/F345/2015 | China | Jiangsu | Chicken | 2015-7-28 | EPI1743138 | OK563151 |
| A/Chicken/Anhui/F598/2015 | China | Anhui | Chicken | 2015-8 | EPI1743146 | OK563154 |
| A/Duck/Anhui/F908/2015 | China | Anhui | Duck | 2015-8 | EPI1743154 | OK563155 |
| A/Chicken/Hunan/F949/2015 | China | Hunan | Chicken | 2015-9-7 | EPI1743162 | OK563156 |
| A/Chicken/Hunan/F998/2015 | China | Hunan | Chicken | 2015-9-7 | EPI1743170 | OK563157 |
| A/Duck/Hunan/F1053/2015 | China | Hunan | Duck | 2015-9-7 | EPI1743178 | OK563235 |
| A/Chicken/Fujian/F1132/2015 | China | Fujian | Chicken | 2015-9 | EPI1743186 | OK563158 |
| A/Duck/Fujian/F1206/2015 | China | Fujian | Duck | 2015-9 | EPI1743194 | OK563159 |
| A/Chicken/Fujian/F1255/2015 | China | Fujian | Chicken | 2015-9 | EPI1743202 | OK563160 |
| A/Chicken/Jiangxi/F1298/2015 | China | Jiangxi | Chicken | 2015-9 | EPI1743210 | OK563161 |
| A/Chicken/Jiangxi/F1303/2015 | China | Jiangxi | Chicken | 2015-9 | EPI1743218 | OK563152 |
| A/Chicken/Jiangxi/F1370/2015 | China | Jiangxi | Chicken | 2015-9 | EPI1743234 | OK563230 |
| A/Chicken/Guangdong/F1650/2015 | China | Guangdong | Chicken | 2015-9-6 | EPI1743242 | OK563153 |
| A/Chicken/Shanghai/F2170/2015 | China | Shanghai | Chicken | 2015-9 | EPI1743570 | OK563149 |
| A/Chicken/Shandong/F2991/2015 | China | Shangdong | Chicken | 2015-7 | EPI1743578 | OK563163 |
| A/Duck/Shandong/F3143/2015 | China | Shangdong | Duck | 2015-7 | EPI1743586 | OK563164 |
| A/Chicken/Guangdong/F4012/2015 | China | Guangdong | Chicken | 2015-9 | EPI1743258 | OK563165 |
| A/Chicken/Guangdong/F4129/2015 | China | Guangdong | Chicken | 2015-9 | EPI1743266 | OK563166 |
| A/Chicken/Guangdong/F4198/2015 | China | Guangdong | Chicken | 2015-9 | EPI1743274 | OK563167 |

Continued Table S1.

| Isolate_Name | Country | Province | Host | Collection_Date | Segment_Ids GISAID | Segment_Ids GeneBank |
|---|---|---|---|---|---|---|
| A/Chicken/Shanghai/G378/2016 | China | Shanghai | Chicken | 2016-11 | EPI1743594 | OK563143 |
| A/Chicken/Jiangxi/G705/2016 | China | Jiangxi | Chicken | 2016-11 | EPI1743282 | OK563142 |
| A/Chicken/Jiangxi/G755/2016 | China | Jiangxi | Chicken | 2016-11 | EPI1743290 | OK563145 |
| A/Chicken/Fujian/G1451/2016 | China | Fujian | Chicken | 2016-11 | EPI1743298 | OK563148 |
| A/Chicken/Guangdong/G1586/2016 | China | Guangdong | Chicken | 2016-11 | EPI1743306 | OK563147 |
| A/Chicken/Guangdong/G1728/2016 | China | Guangdong | Chicken | 2016-11 | EPI1743314 | OK563146 |
| A/Chicken/Guangdong/G1921/2016 | China | Guangdong | Chicken | 2016-11 | EPI1743322 | OK563144 |
| A/Chicken/Shanghai/H96/2017 | China | Shanghai | Chicken | 2017-10-24 | EPI1743602 | OK563138 |
| A/Chicken/Shanghai/H97/2017 | China | Shanghai | Chicken | 2017-10-24 | EPI1743604 | OK563135 |
| A/Chicken/Jiangsu/H224/2017 | China | Jiangsu | Chicken | 2017-10-24 | EPI1743330 | OK563131 |
| A/Chicken/Jiangsu/H491/2017 | China | Jiangsu | Chicken | 2017-10-24 | EPI1743336 | OK563139 |
| A/Chicken/Shanghai/H514/2017 | China | Shanghai | Chicken | 2017-10-24 | EPI1743612 | OK563132 |
| A/Chicken/Guangdong/H938/2017 | China | Guangdong | Chicken | 2017-10-24 | EPI1743343 | OK563140 |
| A/Chicken/Guangdong/H1027/2017 | China | Guangdong | Chicken | 2017-10-24 | EPI1743350 | OK563141 |
| A/Chicken/Hunan/H1114/2017 | China | Hunan | Chicken | 2017-11-21 | EPI1743358 | OK563136 |
| A/Chicken/Jiangxi/H1258/2017 | China | Jiangxi | Chicken | 2017-10-31 | EPI1743366 | OK563137 |
| A/Chicken/Jiangxi/H1301/2017 | China | Jiangxi | Chicken | 2017-10-31 | EPI1743374 | OK563134 |
| A/Chicken/Anhui/H1517/2017 | China | Anhui | Chicken | 2017-10-31 | EPI1743382 | OK563133 |
| A/Chicken/Fuzhou/I25/2018 | China | Fujian | Chicken | 2018-09 | EPI1743384 | OK563127 |
| A/Chicken/Fuzhou/I69/2018 | China | Fujian | Chicken | 2018-09 | EPI1743386 | OK563125 |
| A/Chicken/Fuzhou/I73/2018 | China | Fujian | Chicken | 2018-09 | PI1743388 | OK563128 |
| A/Chicken/Fuzhou/I84/2018 | China | Fujian | Chicken | 2018-09 | PI1743390 | OK563123 |
| A/Chicken/Fuzhou/I92/2018 | China | Fujian | Chicken | 2018-09 | EPI1743392 | OK563129 |
| A/Chicken/Changsha/I96/2018 | China | Hunan | Chicken | 2018-09 | EPI1743614 | OK563130 |
| A/Chicken/Changsha/I97/2018 | China | Hunan | Chicken | 2018-09 | PI1743616 | OK563124 |
| A/Chicken/Changsha/I112/2018 | China | Hunan | Chicken | 2018-09 | EPI1743618 | OK563122 |
| A/Chicken/Nanjing/I256/2018 | China | Jiangsu | Chicken | 2018-09 | EPI1743394 | OK563126 |

Details about nucleotide sequences of H9N2 isolated in Southern China from 2013 to 2018 in this research.

Table S2. Selected H9N2 strains and HI titers of Cross-antigenic Assay

| | | Anti-serum>>>>>> | HI titers[1] | | | | | | | | | | | | | | | | | | | | | | | | | | | | | | | | |
|---|---|---|---|---|---|---|---|---|---|---|---|---|---|---|---|---|---|---|---|---|---|---|---|---|---|---|---|---|---|---|---|---|---|---|---|
| NO. | Abbr. | Antigen | I256 | I112 | I84 | I97 | I69 | I25 | H514 | H224 | H1114 | H1517 | G1728 | G705 | G1451 | G1586 | G378 | G1921 | F2170 | E552 | E2290 | D345 | D2641 | H1301 | H1258 | H97 | G755 | F1650 | D1021 | A2093 | B445 | B469 | 441 | 2106 | F98 |
| 1 | I256 | A/Chicken/Nanjing/I256/2018 | 2048 | 256 | 512 | 1024 | 2048 | 256 | 4096 | 2048 | 1024 | 4096 | 256 | 1024 | 4096 | 1024 | 1024 | 512 | 256 | 512 | 256 | 512 | 1024 | 256 | 128 | 256 | 1024 | 512 | 1024 | 16 | 256 | 64 | 64 | 256 | 32 |
| 2 | I112 | A/Chicken/Changsha/I112/2018 | 512 | 128 | 128 | 256 | 512 | 64 | 512 | 512 | 128 | 512 | 32 | 128 | 1024 | 256 | 128 | 64 | 64 | 128 | 128 | 128 | 1024 | 64 | 64 | 32 | 128 | 128 | 256 | 8 | 64 | 8 | 16 | 32 | 32 |
| 3 | I84 | A/Chicken/Fuzhou/I84/2018 | 512 | 32 | 1024 | 128 | 512 | 128 | 512 | 256 | 64 | 1024 | 64 | 256 | 1024 | 256 | 128 | 128 | 128 | 128 | 128 | 256 | 128 | 128 | 128 | 128 | 256 | 64 | 128 | 8 | 32 | 8 | 32 | 64 | 32 |
| 4 | I97 | A/Chicken/Changsha/I97/2018 | 1024 | 128 | 128 | 512 | 1024 | 64 | 1024 | 512 | 256 | 1024 | 64 | 256 | 2048 | 256 | 256 | 64 | 128 | 256 | 128 | 256 | 128 | 64 | 64 | 64 | 256 | 128 | 256 | 16 | 32 | 8 | 16 | 64 | 32 |
| 5 | I69 | A/Chicken/Fuzhou/I69/2018 | 256 | 128 | 128 | 256 | 4096 | 512 | 1024 | 1024 | 64 | 512 | 512 | 256 | 2048 | 256 | 128 | 256 | 128 | 512 | 256 | 512 | 512 | 64 | 128 | 64 | 512 | 128 | 1024 | 32 | 128 | 16 | 32 | 64 | 128 |
| 6 | I25 | A/Chicken/Fuzhou/I25/2018 | 256 | 128 | 256 | 128 | 4096 | 4096 | 1024 | 2048 | 64 | 512 | 512 | 1024 | 2048 | 1024 | 128 | 2048 | 256 | 1024 | 256 | 1024 | 1024 | 256 | 128 | 64 | 1024 | 128 | 1024 | 16 | 128 | 16 | 32 | 128 | 128 |
| 7 | H514 | A/Chicken/Shanghai/H514/2017 | 1024 | 128 | 256 | 512 | 2048 | 256 | 2048 | 1024 | 256 | 1024 | 256 | 256 | 4096 | 512 | 256 | 256 | 128 | 512 | 512 | 256 | 1024 | 256 | 128 | 256 | 1024 | 128 | 1024 | 16 | 256 | 32 | 64 | 256 | 64 |
| 8 | H224 | A/Chicken/Jiangsu/H224/2017 | 128 | 32 | 64 | 128 | 2048 | 512 | 1024 | 1024 | 64 | 512 | 256 | 256 | 2048 | 128 | 64 | 128 | 128 | 512 | 128 | 256 | 256 | 64 | 128 | 64 | 512 | 64 | 512 | 16 | 64 | 16 | 16 | 64 | 128 |
| 9 | H1114 | A/Chicken/Hunan/H1114/2017 | 4096 | 512 | 512 | 2048 | 4096 | 256 | 2048 | 2048 | 2048 | 4096 | 256 | 1024 | 4096 | 1024 | 2048 | 512 | 512 | 1024 | 512 | 1024 | 2048 | 128 | 128 | 512 | 1024 | 512 | 1024 | 64 | 256 | 32 | 64 | 256 | 64 |
| 10 | H1517 | A/Chicken/Anhui/H1517/2017 | 2048 | 256 | 512 | 1024 | 2048 | 256 | 2048 | 512 | 512 | 4096 | 128 | 1024 | 4096 | 1024 | 2048 | 512 | 256 | 512 | 512 | 512 | 1024 | 128 | 64 | 256 | 1024 | 256 | 512 | 16 | 512 | 64 | 64 | 256 | 16 |
| 11 | G1728 | A/Chicken/Guangdong/G1728/2016 | 256 | 32 | 64 | 128 | 4096 | 512 | 1024 | 1024 | 64 | 512 | 256 | 512 | 4096 | 128 | 128 | 128 | 128 | 512 | 256 | 512 | 256 | 128 | 128 | 128 | 512 | 64 | 512 | 16 | 64 | 16 | 32 | 64 | 128 |
| 12 | G705 | A/Chicken/Jiangxi/G705/2016 | 512 | 64 | 128 | 256 | 2048 | 256 | 512 | 512 | 64 | 512 | 128 | 8192 | 2048 | 512 | 256 | 128 | 256 | 256 | 512 | 256 | 2048 | 64 | 128 | 128 | 512 | 256 | 256 | 4 | 64 | 8 | 32 | 128 | 32 |
| 13 | G1451 | A/Chicken/Fujian/G1451/2016 | 512 | 64 | 128 | 512 | 4096 | 512 | 2048 | 1024 | 128 | 1024 | 128 | 512 | 8192 | 256 | 128 | 256 | 256 | 512 | 128 | 512 | 512 | 512 | 256 | 256 | 1024 | 256 | 2048 | 16 | 256 | 64 | 64 | 256 | 256 |
| 14 | G1586 | A/Chicken/Guangdong/G1586/2016 | 1024 | 256 | 256 | 512 | 2048 | 512 | 2048 | 512 | 256 | 2048 | 128 | 1024 | 2048 | 2048 | 512 | 2048 | 256 | 512 | 256 | 1024 | 1024 | 128 | 64 | 128 | 512 | 256 | 1024 | 16 | 128 | 16 | 32 | 128 | 256 |
| 15 | G378 | A/Chicken/Shanghai/G378/2016 | 2048 | 512 | 512 | 1024 | 4096 | 256 | 2048 | 1024 | 1024 | 4096 | 256 | 1024 | 4096 | 1024 | 2048 | 512 | 256 | 512 | 512 | 1024 | 1024 | 128 | 128 | 256 | 2048 | 512 | 1024 | 8 | 256 | 32 | 64 | 256 | 32 |
| 16 | G1921 | A/Chicken/Guangdong/G1921/2016 | 512 | 128 | 256 | 256 | 1024 | 256 | 2048 | 256 | 128 | 1024 | 64 | 512 | 2048 | 512 | 256 | 2048 | 128 | 512 | 512 | 512 | 256 | 256 | 256 | 128 | 1024 | 128 | 1024 | 16 | 128 | 32 | 32 | 128 | 256 |
| 17 | F2170 | A/Chicken/Shanghai/F2170/2015 | 256 | 64 | 256 | 256 | 4096 | 1024 | 1024 | 1024 | 64 | 1024 | 512 | 2048 | 4096 | 512 | 128 | 1024 | 512 | 1024 | 1024 | 1024 | 2048 | 128 | 128 | 128 | 1024 | 256 | 2048 | 16 | 128 | 8 | 32 | 256 | 512 |
| 18 | E552 | A/Duck/Zhejiang/E552/2014 | 1024 | 256 | 512 | 1024 | 4096 | 2048 | 2048 | 2048 | 256 | 2048 | 1024 | 2048 | 8192 | 1024 | 512 | 512 | 512 | 1024 | 1024 | 1024 | 4096 | 256 | 256 | 512 | 2048 | 512 | 2048 | 16 | 512 | 64 | 64 | 512 | 512 |
| 19 | E2290 | A/Chicken/Anhui/E2290/2014 | 64 | 16 | 32 | 64 | 1024 | 64 | 512 | 128 | 16 | 64 | 16 | 4096 | 1024 | 512 | 16 | 16 | 16 | 256 | 4096 | 256 | 4096 | 32 | 64 | 128 | 1024 | 256 | 2048 | 4 | 512 | 32 | 256 | 512 | 128 |
| 20 | D345 | A/Chicken/Zhejiang/D345/2013 | 256 | 64 | 128 | 128 | 4096 | 1024 | 1024 | 1024 | 64 | 1024 | 256 | 1024 | 4096 | 512 | 64 | 1024 | 256 | 512 | 1024 | 1024 | 2048 | 128 | 128 | 64 | 512 | 256 | 2048 | 16 | 64 | 16 | 32 | 128 | 256 |
| 21 | D2641 | A/Chicken/Guangdong/D2641/2013 | 128 | 32 | 64 | 128 | 2048 | 128 | 512 | 128 | 32 | 64 | 32 | 4096 | 1024 | 512 | 32 | 32 | 16 | 256 | 4096 | 256 | 4096 | 64 | 128 | 128 | 1024 | 256 | 2048 | 8 | 512 | 32 | 256 | 512 | 256 |
| 22 | H1301 | A/Chicken/Jiangxi/H1301/2017 | 128 | 32 | 32 | 32 | 256 | 64 | 128 | 32 | 8 | 64 | 32 | 32 | 1024 | 32 | 32 | 64 | 16 | 64 | 256 | 64 | 256 | 1024 | 1024 | 512 | 2048 | 1024 | 4096 | 128 | 1024 | 256 | 512 | 1024 | 1024 |
| 23 | H1258 | A/Chicken/Jiangxi/H1258/2017 | 32 | 16 | 32 | 16 | 128 | 16 | 64 | 32 | 8 | 16 | 16 | 16 | 512 | 16 | 8 | 32 | 8 | 32 | 128 | 64 | 128 | 1024 | 512 | 256 | 2048 | 512 | 2048 | 128 | 512 | 128 | 256 | 1024 | 1024 |
| 24 | H97 | A/Chicken/Shanghai/H97/2017 | 64 | 16 | 32 | 32 | 128 | 32 | 128 | 32 | 8 | 32 | 16 | 32 | 512 | 32 | 16 | 64 | 8 | 32 | 128 | 64 | 128 | 512 | 512 | 512 | 2048 | 1024 | 4096 | 128 | 1024 | 256 | 512 | 1024 | 1024 |
| 25 | G755 | A/Chicken/Jiangxi/G755/2016 | 128 | 32 | 32 | 32 | 256 | 64 | 128 | 64 | 8 | 64 | 16 | 64 | 1024 | 32 | 16 | 64 | 32 | 64 | 256 | 64 | 256 | 1024 | 1024 | 512 | 4096 | 1024 | 4096 | 64 | 1024 | 256 | 512 | 1024 | 2048 |
| 26 | F1650 | A/Chicken/Guangdong/F1650/2015 | 16 | 16 | 16 | 16 | 64 | 32 | 64 | 16 | 2 | 16 | 16 | 16 | 256 | 16 | 8 | 32 | 16 | 32 | 128 | 32 | 128 | 512 | 512 | 256 | 2048 | 512 | 2048 | 64 | 512 | 128 | 256 | 512 | 1024 |
| 27 | D1021 | A/Chicken/Hunan/D1021/2013 | 32 | 32 | 32 | 16 | 128 | 32 | 64 | 32 | 4 | 16 | 16 | 32 | 256 | 32 | 16 | 64 | 16 | 32 | 256 | 32 | 128 | 512 | 512 | 256 | 2048 | 512 | 4096 | 128 | 1024 | 64 | 256 | 1024 | 1024 |
| 28 | A2093 | A/Chicken/Jiangsu/A2093/2011 | 32 | 128 | 64 | 32 | 128 | 32 | 256 | 64 | 2 | 128 | 16 | 128 | 256 | 32 | 64 | 128 | 128 | 64 | 512 | 64 | 256 | 512 | 256 | 128 | 4096 | 512 | 4096 | 256 | 256 | 64 | 512 | 1024 | 2048 |
| 29 | B445 | A/chicken/Hunan/B445/2011# | 128 | 32 | 32 | 32 | 512 | 64 | 256 | 64 | 8 | 128 | 16 | 64 | 2048 | 64 | 32 | 128 | 8 | 64 | 512 | 256 | 256 | 2048 | 1024 | 1024 | 4096 | 2048 | 8192 | 512 | 2048 | 1024 | 2048 | 4096 | 4096 |
| 30 | B469 | A/chicken/Shanghai/B469/2011# | 64 | 64 | 32 | 256 | 64 | 256 | 64 | 8 | 128 | 16 | 64 | 2048 | 64 | 32 | 64 | 16 | 128 | 256 | 256 | 128 | 2048 | 1024 | 1024 | 2048 | 1024 | 8192 | 128 | 1024 | 1024 | 1024 | 1024 | 2048 | 2048 |
| 31 | 441 | A/Chicken/Shanghai/441/2009# | 64 | 64 | 64 | 32 | 256 | 64 | 256 | 32 | 4 | 128 | 8 | 32 | 2048 | 64 | 32 | 128 | 16 | 128 | 512 | 256 | 256 | 4096 | 1024 | 1024 | 4096 | 2048 | 8192 | 256 | 4096 | 1024 | 2048 | 4096 | 2048 |
| 32 | 2106 | A/chicken/Hunan/2106/2009# | 128 | 64 | 128 | 64 | 512 | 128 | 256 | 64 | 8 | 128 | 16 | 128 | 2048 | 64 | 64 | 128 | 32 | 128 | 512 | 256 | 256 | 2048 | 1024 | 1024 | 4096 | 2048 | 8192 | 512 | 4096 | 1024 | 2048 | 4096 | 2048 |
| 33 | F98 | A/Chicken/Shanghai/F/1998 | 8 | 4 | 4 | 4 | 8 | 8 | 8 | 8 | 1 | 8 | 1 | 8 | 64 | 8 | 8 | 8 | 8 | 8 | 64 | 8 | 16 | 64 | 256 | 32 | 256 | 64 | 1024 | 16 | 256 | 16 | 64 | 256 | 1024 |

NOTE: The bold line grouped the virus strains basing on the time-scaled tree. 1 The background color depth were based on the value of HI titers, red was the high HI titer, indicating close antigenicity; light yellow was low HI titers indicating big distance in activity.

TableS3.The antigenic distances

| Strains | I256 | I112 | I84 | I97 | I69 | I25 | H514 | H224 | H1114 | H1517 | G1728 | G705 | G1451 | G1586 | G378 | G1921 | F2170 | E552 | E2290 | D345 | D2641 | H1301 | H1258 | H97 | G755 | F1650 | D1021 | A2093 | B445 | B469 | 441 | 2106 | F98* |
|---|---|---|---|---|---|---|---|---|---|---|---|---|---|---|---|---|---|---|---|---|---|---|---|---|---|---|---|---|---|---|---|---|---|
| **I256** | 0 | 2.562672 | 5.131206 | 1.393596 | 5.919718 | 7.881337 | 4.472925 | 5.312156 | 1.542524 | 2.405899 | 6.922 | 7.703483 | 5.996075 | 5.053535 | 2.421182 | 7.17391 | 4.832598 | 5.435165 | 8.265712 | 5.432955 | 7.0213 | 9.075241 | 9.345873 | 8.231075 | 8.85059 | 8.757525 | 9.324753 | 8.865774 | 8.942386 | 8.965403 | 9.080415 | 9.050292 | 9.368326 |
| **I112** | 2.562672 | 0 | 5.314532 | 2.55292 | 5.568363 | 7.328958 | 4.245524 | 5.137418 | 2.760476 | 2.40868 | 6.300339 | 7.835448 | 5.980995 | 4.364124 | 2.586355 | 6.50359 | 4.755528 | 5.244807 | 8.285411 | 4.650438 | 6.963964 | 8.981761 | 9.320452 | 8.317152 | 8.393333 | 8.658722 | 9.171505 | 8.708638 | 8.895732 | 8.91596 | 8.976053 | 8.935262 | 9.268199 |
| **I84** | 5.131206 | 5.314532 | 0 | 5.197315 | 6.452434 | 7.083417 | 5.436593 | 6.006364 | 5.729935 | 4.52018 | 6.324828 | 7.815711 | 6.325019 | 5.636071 | 5.234084 | 6.834875 | 5.307711 | 5.921823 | 8.323905 | 5.70759 | 7.242346 | 9.107316 | 9.41676 | 8.609893 | 9.028128 | 9.063067 | 9.439288 | 9.021588 | 9.026057 | 9.019331 | 9.142646 | 9.123295 | 9.468001 |
| **I97** | 1.393596 | 2.55292 | 5.197315 | 0 | 5.347661 | 7.502283 | 3.955802 | 4.671734 | 2.214893 | 2.450482 | 6.089312 | 7.544501 | 5.1145 | 4.886915 | 2.850419 | 7.15584 | 4.125076 | 4.806733 | 8.106401 | 5.016207 | 6.493923 | 9.158624 | 9.441496 | 8.252934 | 8.855918 | 8.846322 | 9.35582 | 8.989928 | 9.011616 | 9.065073 | 9.195414 | 9.133231 | 9.444972 |
| **I69** | 5.919718 | 5.568363 | 6.452434 | 5.347661 | 0 | 5.159209 | 4.32314 | 2.849926 | 6.021592 | 5.368188 | 3.90788 | 6.853161 | 3.923171 | 5.337656 | 6.099561 | 6.083674 | 3.696435 | 2.840586 | 7.56688 | 3.089213 | 5.932548 | 9.607399 | 9.968952 | 9.276676 | 9.527074 | 9.566021 | 9.688081 | 9.523878 | 9.570713 | 9.537896 | 9.656944 | 9.646147 | 9.897036 |
| **I25** | 7.881337 | 7.328958 | 7.083417 | 7.502283 | 5.159209 | 0 | 6.650762 | 4.23779 | 7.998637 | 7.492371 | 4.292045 | 7.550081 | 6.339336 | 6.012235 | 7.946663 | 5.026936 | 5.636023 | 4.406349 | 8.072407 | 4.909443 | 6.773855 | 8.783775 | 9.003262 | 8.782658 | 8.684565 | 8.903125 | 8.852719 | 8.854365 | 8.839197 | 8.828113 | 8.92728 | 8.886583 | 8.952108 |
| **H514** | 4.472925 | 4.245524 | 5.436593 | 3.955802 | 4.32314 | 6.650762 | 0 | 4.43699 | 5.231507 | 3.651252 | 5.467397 | 7.628805 | 3.936131 | 4.032269 | 4.789201 | 5.349559 | 4.261779 | 3.897711 | 8.042498 | 4.115855 | 6.681048 | 9.536957 | 9.977535 | 9.078128 | 9.438988 | 9.575686 | 9.815564 | 9.526972 | 9.526636 | 9.482756 | 9.660624 | 9.637542 | 9.961707 |
| **H224** | 5.312156 | 5.137418 | 6.006364 | 4.671734 | 2.849926 | 4.23779 | 4.43699 | 0 | 5.435653 | 5.323103 | 3.235209 | 7.442363 | 4.344656 | 5.315735 | 6.03424 | 6.063722 | 3.391681 | 2.273741 | 8.236392 | 3.615341 | 6.223734 | 9.509087 | 9.835807 | 9.041062 | 9.392615 | 9.487473 | 9.693737 | 9.422799 | 9.516732 | 9.481874 | 9.639348 | 9.596759 | 9.79809 |
| **H1114** | 1.542524 | 2.760476 | 5.729935 | 2.214893 | 6.021592 | 7.998637 | 5.231507 | 5.435653 | 0 | 3.309781 | 7.147486 | 7.873318 | 6.446914 | 5.673663 | 2.986068 | 7.420173 | 5.218179 | 5.674899 | 8.1677 | 5.603454 | 7.131749 | 8.869211 | 9.079233 | 8.031519 | 8.572084 | 8.504412 | 9.035475 | 8.64102 | 8.755705 | 8.783591 | 8.865764 | 8.835121 | 9.106218 |
| **H1517** | 2.405899 | 2.40868 | 4.52018 | 2.450482 | 5.368188 | 7.492371 | 3.651252 | 5.323103 | 3.309781 | 0 | 6.39834 | 7.71423 | 5.365906 | 4.281339 | 1.649573 | 6.558804 | 4.412411 | 5.098631 | 8.273432 | 4.588504 | 6.963768 | 9.234261 | 9.578149 | 8.530828 | 8.92418 | 8.97151 | 9.479434 | 9.114321 | 9.101721 | 9.124286 | 9.27609 | 9.233395 | 9.538811 |
| **G1728** | 6.922 | 6.300339 | 6.324828 | 6.089312 | 3.90788 | 4.292045 | 5.467397 | 3.235209 | 7.147486 | 6.39834 | 0 | 7.342109 | 4.651798 | 5.927761 | 7.115933 | 6.427404 | 4.015395 | 3.367898 | 7.98302 | 4.351723 | 5.875337 | 9.198454 | 9.377264 | 8.749625 | 8.889199 | 9.123379 | 9.208668 | 9.179682 | 9.138553 | 9.173451 | 9.313846 | 9.21874 | 9.288716 |
| **G705** | 7.703483 | 7.835448 | 7.815711 | 7.544501 | 6.853161 | 7.550081 | 7.628805 | 7.442363 | 7.873318 | 7.71423 | 7.342109 | 0 | 7.524424 | 6.652424 | 7.692876 | 7.854345 | 6.721169 | 7.121311 | 5.31053 | 7.255276 | 4.414254 | 9.135365 | 9.308626 | 8.965614 | 9.030384 | 8.950766 | 9.070756 | 9.075508 | 8.897514 | 8.995736 | 8.900234 | 8.923358 | 9.208634 |
| **G1451** | 5.996075 | 5.980995 | 6.325019 | 5.1145 | 3.923171 | 6.339336 | 3.936131 | 4.344656 | 6.446914 | 5.365906 | 4.651798 | 7.524424 | 0 | 6.118257 | 6.24482 | 7.061541 | 4.193409 | 4.352785 | 8.064987 | 4.661316 | 6.388082 | 8.571855 | 8.974952 | 7.986393 | 8.62412 | 8.62866 | 8.751944 | 8.806002 | 8.652698 | 8.500465 | 8.762905 | 8.681916 | 9.121537 |
| **G1586** | 5.053535 | 4.364124 | 5.636071 | 4.886915 | 5.337656 | 6.012235 | 4.032269 | 5.315735 | 5.673663 | 4.281339 | 5.927761 | 6.652424 | 6.118257 | 0 | 5.105069 | 4.381258 | 4.675081 | 4.633572 | 7.332592 | 3.696977 | 5.802246 | 9.441115 | 9.850815 | 9.037984 | 9.292782 | 9.285489 | 9.576583 | 9.381043 | 9.30172 | 9.34351 | 9.429806 | 9.411379 | 9.691357 |
| **G378** | 2.421182 | 2.586355 | 5.234084 | 2.850419 | 6.099561 | 7.946663 | 4.789201 | 6.03424 | 2.986068 | 1.649573 | 7.115933 | 7.692876 | 6.24482 | 5.105069 | 0 | 7.22907 | 5.390295 | 5.936294 | 8.194701 | 5.574939 | 7.242847 | 8.926354 | 9.192691 | 8.177693 | 8.4896 | 8.56844 | 9.137526 | 8.755638 | 8.732102 | 8.800014 | 8.90771 | 8.868982 | 9.182034 |
| **G1921** | 7.17391 | 6.50359 | 6.834875 | 7.15584 | 6.083674 | 5.026936 | 5.349559 | 6.063722 | 7.420173 | 6.558804 | 6.427404 | 7.854345 | 7.061541 | 4.381258 | 7.22907 | 0 | 5.800921 | 5.030164 | 8.254384 | 4.374949 | 7.534138 | 8.901738 | 9.183836 | 9.016387 | 8.938791 | 9.028341 | 9.033042 | 8.908635 | 9.010741 | 8.940295 | 9.038803 | 9.042307 | 9.081208 |
| **F2170** | 4.832598 | 4.755528 | 5.307711 | 4.125076 | 3.696435 | 5.636023 | 4.261779 | 3.391681 | 5.218179 | 4.412411 | 4.015395 | 6.721169 | 4.193409 | 4.675081 | 5.390295 | 5.800921 | 0 | 2.681953 | 8.124291 | 3.151879 | 5.876411 | 9.501548 | 9.813192 | 8.953026 | 9.149506 | 9.341167 | 9.568465 | 9.31513 | 9.48394 | 9.48367 | 9.59839 | 9.524769 | 9.623681 |
| **E552** | 5.435165 | 5.244807 | 5.921823 | 4.806733 | 2.840586 | 4.406349 | 3.897711 | 2.273741 | 5.674899 | 5.098631 | 3.367898 | 7.121311 | 4.352785 | 4.633572 | 5.936294 | 5.030164 | 2.681953 | 0 | 7.668952 | 2.738034 | 5.790435 | 9.465177 | 9.862179 | 9.041633 | 9.371167 | 9.479878 | 9.599457 | 9.453132 | 9.4348 | 9.420637 | 9.578361 | 9.535566 | 9.803974 |
| **E2290** | 8.265712 | 8.285411 | 8.323905 | 8.106401 | 7.56688 | 8.072407 | 8.042498 | 8.236392 | 8.1677 | 8.273432 | 7.98302 | 5.31053 | 8.064987 | 7.332592 | 8.194701 | 8.254384 | 8.124291 | 7.668952 | 0 | 7.720218 | 3.504082 | 8.595847 | 8.798185 | 8.487513 | 8.258641 | 8.402916 | 8.148384 | 8.475178 | 8.200649 | 8.421267 | 8.117975 | 8.165836 | 8.587564 |
| **D345** | 5.432955 | 4.650438 | 5.70759 | 5.016207 | 3.089213 | 4.909443 | 4.115855 | 3.615341 | 5.603454 | 4.588504 | 4.351723 | 7.255276 | 4.661316 | 3.696977 | 5.574939 | 4.374949 | 3.151879 | 2.738034 | 7.720218 | 0 | 5.990248 | 9.138928 | 9.590372 | 8.758736 | 9.078244 | 9.05803 | 9.226193 | 9.096303 | 9.158444 | 9.012624 | 9.1867 | 9.152861 | 9.520056 |
| **D2641** | 7.0213 | 6.963964 | 7.242346 | 6.493923 | 5.932548 | 6.773855 | 6.681048 | 6.223734 | 7.131749 | 6.963768 | 5.875337 | 4.414254 | 6.388082 | 5.802246 | 7.242847 | 7.534138 | 5.876411 | 5.790435 | 3.504082 | 5.990248 | 0 | 9.278386 | 9.49734 | 8.811321 | 8.887164 | 8.991307 | 8.990632 | 9.191973 | 8.937074 | 9.128872 | 9.006251 | 8.976326 | 9.333152 |
| **H1301** | 9.075241 | 8.981761 | 9.107316 | 9.158624 | 9.607399 | 8.783775 | 9.536957 | 9.509087 | 8.869211 | 9.234261 | 9.198454 | 9.135365 | 8.571855 | 9.441115 | 8.926354 | 8.901738 | 9.501548 | 9.465177 | 8.595847 | 9.138928 | 9.278386 | 0 | 3.3751 | 2.875827 | 4.180321 | 2.680892 | 2.69897 | 4.511973 | 2.500152 | 2.407345 | 2.446949 | 2.394141 | 3.943625 |
| **H1258** | 9.345873 | 9.320452 | 9.41676 | 9.441496 | 9.968952 | 9.003262 | 9.977535 | 9.835807 | 9.079233 | 9.578149 | 9.377264 | 9.308626 | 8.974952 | 9.850815 | 9.192691 | 9.183836 | 9.813192 | 9.862179 | 8.798185 | 9.590372 | 9.49734 | 3.3751 | 0 | 3.408366 | 3.632254 | 3.343648 | 3.095849 | 4.227214 | 4.11268 | 3.18072 | 3.446859 | 3.386343 | 4.012515 |
| **H97** | 8.231075 | 8.317152 | 8.609893 | 8.252934 | 9.276676 | 8.782658 | 9.078128 | 9.041062 | 8.031519 | 8.530828 | 8.749625 | 8.965614 | 7.986393 | 9.037984 | 8.177693 | 9.016387 | 8.953026 | 9.041633 | 8.487513 | 8.758736 | 8.811321 | 2.875827 | 3.408366 | 0 | 4.276551 | 2.02648 | 2.386822 | 4.408336 | 2.600765 | 2.286919 | 2.822079 | 2.768005 | 3.624888 |
| **G755** | 8.85059 | 8.393333 | 9.028128 | 8.855918 | 9.527074 | 8.684565 | 9.438988 | 9.392615 | 8.572084 | 8.92418 | 8.889199 | 9.030384 | 8.62412 | 9.292782 | 8.4896 | 8.938791 | 9.149506 | 9.371167 | 8.258641 | 9.078244 | 8.887164 | 4.180321 | 3.632254 | 4.276551 | 0 | 3.600799 | 3.676147 | 3.631975 | 4.613508 | 4.360013 | 3.665238 | 3.536102 | 3.992078 |
| **F1650** | 8.757525 | 8.658722 | 9.063067 | 8.846322 | 9.566021 | 8.903125 | 9.575686 | 9.487473 | 8.504412 | 8.97151 | 9.123379 | 8.950766 | 8.62866 | 9.285489 | 8.56844 | 9.028341 | 9.341167 | 9.479878 | 8.402916 | 9.05803 | 8.991307 | 2.680892 | 3.343648 | 2.02648 | 3.600799 | 0 | 2.11375 | 3.711784 | 2.088954 | 2.675657 | 2.06957 | 2.070304 | 2.919243 |
| **D1021** | 9.324753 | 9.171505 | 9.439288 | 9.35582 | 9.688081 | 8.852719 | 9.815564 | 9.693737 | 9.035475 | 9.479434 | 9.208668 | 9.070756 | 8.751944 | 9.576583 | 9.137526 | 9.033042 | 9.568465 | 9.599457 | 8.148384 | 9.226193 | 8.990632 | 2.69897 | 3.095849 | 2.386822 | 3.676147 | 2.11375 | 0 | 3.847178 | 2.501356 | 2.322761 | 2.348812 | 2.306185 | 2.674347 |
| **A2093** | 8.865774 | 8.708638 | 9.021588 | 8.989928 | 9.523878 | 8.854365 | 9.526972 | 9.422799 | 8.64102 | 9.114321 | 9.179682 | 9.075508 | 8.806002 | 9.381043 | 8.755638 | 8.908635 | 9.31513 | 9.453132 | 8.475178 | 9.096303 | 9.191973 | 4.511973 | 4.227214 | 4.408336 | 3.631975 | 3.711784 | 3.847178 | 0 | 4.593559 | 3.576248 | 2.689673 | 2.594544 | 4.297972 |
| **B445** | 8.942386 | 8.895732 | 9.026057 | 9.011616 | 9.570713 | 8.839197 | 9.526636 | 9.516732 | 8.755705 | 9.101721 | 9.138553 | 8.897514 | 8.652698 | 9.30172 | 8.732102 | 9.010741 | 9.48394 | 9.4348 | 8.200649 | 9.158444 | 8.937074 | 2.500152 | 4.11268 | 2.600765 | 4.613508 | 2.088954 | 2.501356 | 4.593559 | 0 | 2.884273 | 2.601968 | 2.595429 | 3.786211 |
| **B469** | 8.965403 | 8.91596 | 9.019331 | 9.065073 | 9.537896 | 8.828113 | 9.482756 | 9.481874 | 8.783591 | 9.124286 | 9.173451 | 8.995736 | 8.500465 | 9.34351 | 8.800014 | 8.940295 | 9.48367 | 9.420637 | 8.421267 | 9.012624 | 9.128872 | 2.407345 | 3.18072 | 2.286919 | 4.360013 | 2.675657 | 2.322761 | 3.576248 | 2.884273 | 0 | 1.717219 | 1.839927 | 3.863331 |
| **441** | 9.080415 | 8.976053 | 9.142646 | 9.195414 | 9.656944 | 8.92728 | 9.660624 | 9.639348 | 8.865764 | 9.27609 | 9.313846 | 8.900234 | 8.762905 | 9.429806 | 8.90771 | 9.038803 | 9.59839 | 9.578361 | 8.117975 | 9.1867 | 9.006251 | 2.446949 | 3.446859 | 2.822079 | 3.665238 | 2.06957 | 2.348812 | 2.689673 | 2.601968 | 1.717219 | 0 | 0.639992 | 3.529127 |
| **2106** | 9.050292 | 8.935262 | 9.123295 | 9.133231 | 9.646147 | 8.886583 | 9.637542 | 9.596759 | 8.835121 | 9.233395 | 9.21874 | 8.923358 | 8.681916 | 9.411379 | 8.868982 | 9.042307 | 9.524769 | 9.535566 | 8.165836 | 9.152861 | 8.976326 | 2.394141 | 3.386343 | 2.768005 | 3.536102 | 2.070304 | 2.306185 | 2.594544 | 2.595429 | 1.839927 | 0.639992 | 0 | 3.517796 |
| **F98*** | 9.368326 | 9.268199 | 9.468001 | 9.444972 | 9.897036 | 8.952108 | 9.961707 | 9.79809 | 9.106218 | 9.538811 | 9.288716 | 9.208634 | 9.121537 | 9.691357 | 9.182034 | 9.081208 | 9.623681 | 9.803974 | 8.587564 | 9.520056 | 9.333152 | 3.943625 | 4.012515 | 3.624888 | 3.992078 | 2.919243 | 2.674347 | 4.297972 | 3.786211 | 3.863331 | 3.529127 | 3.517796 | 0 |

* the vaccine strain.
